# Design of asymmetric electronic spring for stabilizing selective seawater oxidation

**DOI:** 10.1093/nsr/nwag091

**Published:** 2026-02-10

**Authors:** Lili Guo, Chao Feng, Jingqi Chi, Tianrong Zhan, Zekun Wang, Hailing Guo, Zhi Su, Xiaobin Liu, Zexing Wu, Jianping Lai, Lei Wang

**Affiliations:** Key Laboratory of Eco-chemical Engineering, International Science and Technology Cooperation Base of Eco-chemical Engineering and Green Manufacturing, College of Chemical Engineering, Qingdao University of Science and Technology, Qingdao 266042, China; State Key Laboratory of Heavy Oil Processing, College of Chemical Engineering, China University of Petroleum (East China), Qingdao 266580, China; College of Chemical and Biological Engineering, Shandong University of Science and Technology, Qingdao 266590, China; Key Laboratory of Eco-chemical Engineering, International Science and Technology Cooperation Base of Eco-chemical Engineering and Green Manufacturing, College of Chemical Engineering, Qingdao University of Science and Technology, Qingdao 266042, China; College of Chemistry and Molecular Engineering, Qingdao University of Science and Technology, Qingdao 266042, China; College of Chemistry and Molecular Engineering, Qingdao University of Science and Technology, Qingdao 266042, China; State Key Laboratory of Heavy Oil Processing, College of Chemical Engineering, China University of Petroleum (East China), Qingdao 266580, China; State Key Laboratory of Chemistry and Utilization of Carbon, Based Energy Resources, College of Chemistry, Xinjiang University, Urumqi 830017, China; Key Laboratory of Eco-chemical Engineering, International Science and Technology Cooperation Base of Eco-chemical Engineering and Green Manufacturing, College of Chemical Engineering, Qingdao University of Science and Technology, Qingdao 266042, China; Key Laboratory of Eco-chemical Engineering, International Science and Technology Cooperation Base of Eco-chemical Engineering and Green Manufacturing, College of Chemical Engineering, Qingdao University of Science and Technology, Qingdao 266042, China; College of Chemistry and Molecular Engineering, Qingdao University of Science and Technology, Qingdao 266042, China; Key Laboratory of Eco-chemical Engineering, International Science and Technology Cooperation Base of Eco-chemical Engineering and Green Manufacturing, College of Chemical Engineering, Qingdao University of Science and Technology, Qingdao 266042, China; College of Chemistry and Molecular Engineering, Qingdao University of Science and Technology, Qingdao 266042, China; Key Laboratory of Eco-chemical Engineering, International Science and Technology Cooperation Base of Eco-chemical Engineering and Green Manufacturing, College of Chemical Engineering, Qingdao University of Science and Technology, Qingdao 266042, China; College of Chemistry and Molecular Engineering, Qingdao University of Science and Technology, Qingdao 266042, China

**Keywords:** NiFe-layered double hydroxide, oxygen-evolution reaction, seawater splitting, AEM electrolyser

## Abstract

The core in direct seawater electrolysis lies in developing efficient and corrosion-resistant electrocatalysts to suppress detrimental chloride oxidation reaction. This work introduces an anodic electrocatalyst interface modulation strategy, revealing that NiFeOOH modified with Cr_2_O_3_ forms flexible Cr–O–Ni asymmetric bonds, which function as a dynamic ‘electronic spring’ during the oxygen-evolution reaction (OER). The introduction of Cr_2_O_3_ selectively converts NiFeOOH into the active *β* phase. Under high potentials, the dynamic Cr–O–Ni electronic modulation prevents the overoxidation of Ni active sites, stabilizing them in the highly active +3 oxidation state, and simultaneously promotes the transformation from the adsorbate evolution mechanism (AEM) to the lattice oxygen oxidation mechanism. In alkaline seawater, high-valence Cr acts as a Lewis acid to enhance OH^−^ adsorption while its electrostatic repulsion suppresses Cl^−^ accumulation, significantly boosting OER activity and selectivity. Remarkably, the as-synthesized Cr_2_O_3_–NiFeOOH delivers 1.0 A cm^−2^ at just 1.60 V in alkaline seawater, maintaining exceptional stability for over 500 h, and can even maintain high stability at 500 mA cm^−2^ when deployed in an AEM electrolyser. The Cr_2_O_3_–NiFeOOH anode also achieves 77.9% energy efficiency at 100 mA cm^−2^, producing hydrogen at $0.85 per gasoline gallon equivalent, demonstrating industrial viability for seawater electrolysis.

## INTRODUCTION

The integration of renewable energy with water electrolysis for the production of high-purity hydrogen (H_2_) represents a promising and sustainable solution to mitigate climate change and ensure energy security [1]. Current state-of-the-art water electrolysers, such as proton exchange membrane water electrolysers (PEMWEs), alkaline water electrolysers (AWEs) and anion-exchange-membrane water electrolysers (AEMWEs), typically rely on high-purity water as the feedstock [2]. This reliance, however, is at odds with global water-distribution challenges. Consequently, the use of seawater, which constitutes ∼96.5% of Earth’s water reserves, as a feedstock for electrolysis with minimal pretreatment has emerged as an attractive and feasible alternative. Additionally, seawater, with its high salt concentration (∼0.5 M NaCl), exhibits enhanced electrical conductivity (33.9 mS cm^−1^ at 25°C) relative to pure water, thereby rendering it an ideal medium for electrolysis [3]. Seawater electrolysis, therefore, presents one of the most promising and environmentally benign routes for hydrogen production [4]. However, in comparison with pure-water electrolysis, seawater electrolysis introduces additional complexities, particularly regarding the catalyst requirements. The presence of harmful chloride ions, the toxic effects of impurities and the formation of precipitates necessitate electrocatalysts with high stability and robust activity [5]. Alkaline-seawater electrolysis has emerged as the predominant method for seawater electrolysis, given its ability to obviate the need for continuous alkali supplementation and effectively suppress the anodic chloride oxidation reaction (ClOR). Precious metals, owing to their high intrinsic activity and stability during the oxygen-evolution reaction (OER), have traditionally been favored for OER applications. However, their scarcity and high cost underscore the critical need for the development of non-precious-metal catalysts that are both cost-effective and capable of providing long-term catalytic performance [6]. Among the 3d transition metals, NiFe-based catalysts have garnered significant attention as promising candidates for seawater oxidation, owing to their superior catalytic properties. The crystal structure of Ni(Fe)OOH, which primarily exists in *β*- and *γ*-phases, plays a pivotal role in determining the catalytic activity for the OER. *β*-Ni(Fe)OOH, characterized by a lower Ni oxidation state (+3), has been shown to outperform the *γ*-phase, which features a higher oxidation state (3 + σ). Therefore, it is essential to prevent the overoxidation of Ni during anodic OER processes in order to maintain the stability and efficiency of the catalyst [7,8].

During seawater electrolysis, the inherently corrosive nature of Cl^−^ leads to its oxidation into even more aggressive active chlorine species (Cl_2_/HClO/ClO^−^). Under conditions of elevated current densities and extended reaction durations, the increased adsorption of Cl^−^ makes it difficult to avoid the chlorine-induced etching of metal sites [9]. As a result, the durability of electrodes is predominantly determined by the competitive dynamics between Cl^−^ and OH^−^. Although the construction of anion-rich surfaces, such as those incorporating PO_4_^3−^ or CO_3_^2−^, can electrostatically repel Cl^−^, this approach may also result in the repulsion of OH^−^ [10]. A promising strategy to enhance the selective adsorption of OH^−^ involves the introduction of a hard Lewis-acid layer on the catalyst, which effectively captures surrounding hydroxyl anions (hard Lewis bases) [11]. Among the transition metals, Cr^3+^ stands out as the hardest Lewis acid, characterized by an exceptionally low p*K*_a_ value of 2.05, making it an optimal candidate for the Lewis-acid layer [12]. While there have been limited reports on strategies that combine catalysts with Lewis acids, the dynamic regulation of catalysts to simultaneously achieve high activity and stability during seawater electrolysis remains an unexplored area of research.

**Figure 1.**
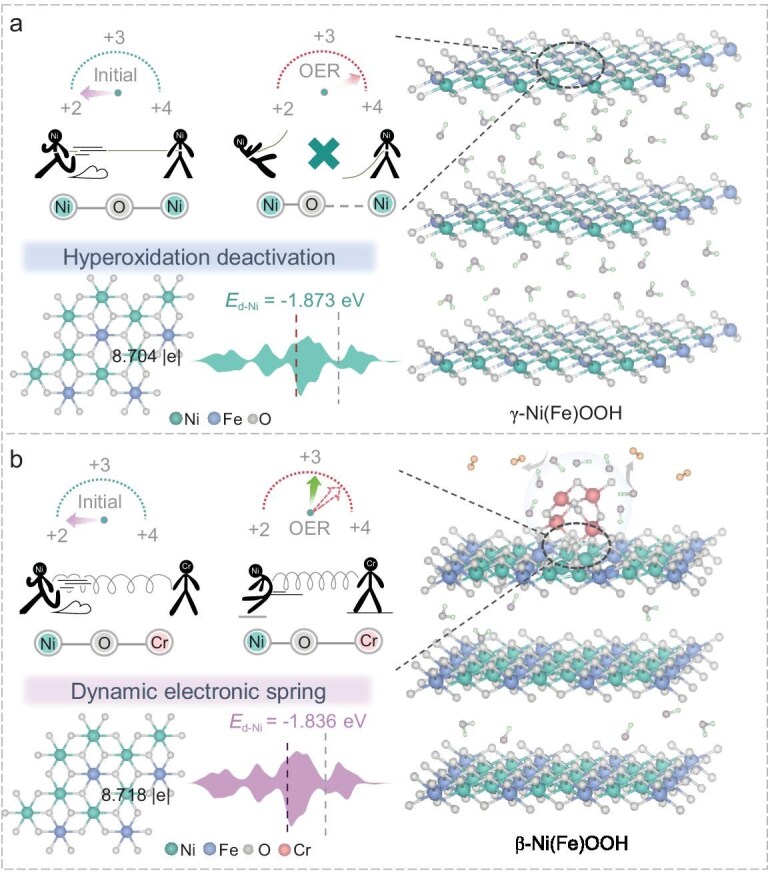
Schematic illustration of the dynamic regulation of the electronic structure inspired by spring-like adjustment, achieved through Cr–O–Ni asymmetric bridging oxygen bonds: (a) NiFeOOH; (b) Cr_2_O_3_–NiFeOOH.

Drawing inspiration from the elastic properties of springs, we propose the design of a novel electrocatalyst that functions akin to an ‘electronic spring’, capable of dynamically adjusting the oxidation state of active sites during the OER process, thus mitigating the risk of overoxidation (Fig. 1a and b). In this work, we combine high-valence Cr species with NiFe-LDH (layered double hydroxide) to form Cr_2_O_3_–NiFe-LDH. The activated catalyst, Cr_2_O_3_–NiFeOOH, features abundant Cr–O–Ni asymmetric active oxygen bridge bonds at the interface. These bridging oxygens act as a dynamic ‘electronic spring’ during the OER process. *In situ* Raman spectroscopy reveals that the catalyst facilitates the transformation of the NiFe substrate into the more active *β*-Ni(Fe)OOH phase at low potentials. Complementary *in situ* synchrotron radiation characterization further confirms a reverse electron-transfer mechanism from Cr to Ni centers under high-potential conditions, stabilizing the active Ni sites in Cr_2_O_3_–NiFe-LDH at the +3 oxidation state throughout the OER process. This reverse electron-transfer mechanism, mediated by Cr, reduces Ni–O coordination and effectively prevents the deleterious effects of overoxidation, thus preserving the catalytic activity. Furthermore, Cr, as a high-valence Lewis acid, enhances the adsorption of OH^−^ on the anode surface and facilitates mass transport, while also mitigating the accumulation of Cl^−^, thereby realizing a stability of >500 h during the OER process in alkaline seawater. When implemented in an adsorbate evolution mechanism (AEM) electrolyser, the Cr_2_O_3_–NiFeOOH||Pt/C catalyst exhibits an energy-conversion efficiency of 77.9% at 100 mA cm^−2^, with a hydrogen-production cost of $0.85 per gasoline gallon equivalent (GGE), which is significantly lower than the US Department of Energy’s 2026 target of $2.00/GGE. This work paves the way for the development of highly active and stable electrocatalysts for seawater electrolysis and OER applications, offering a new strategy for advancing clean hydrogen production.

## RESULTS AND DISCUSSION

### Synthesis and characterization of Cr_2_O_3_–NiFeOOH nanosheets

The *β*- and *γ*-Ni(Fe)OOH phases exhibit similar structural frameworks, distinguished primarily by the larger interlayer spacing in the *γ*-phase [13]. This expanded spacing renders the structure susceptible to collapse under excessive OH^−^ attack during catalytic operation, leading to deactivation. Density functional theory (DFT) calculations were performed to investigate the phase-transition effects induced by Cr_2_O_3_ loading on Ni(Fe)OOH during the OER. Details of the definitions and calculations are given in the Supplementary information, and the structural schematic diagrams of NiFeOOH and Cr_2_O_3_–NiFeOOH are shown in Fig. S1 [14]. Projected density-of-states (DOS) analysis reveals a shift of the Ni d-band center from −1.873 eV in pristine NiFeOOH to −1.836 eV in Cr_2_O_3_–NiFeOOH, indicating that the interfacial orbital hybridization regulates the local electronic structure of the Ni site, which may affect its ability to promote the adsorption and activation of intermediates in the electrolytic seawater reaction [15]. The electron redistribution was confirmed by using Bader charge analysis, with the Ni charge increasing from 8.704 to 8.718 |e| upon Cr_2_O_3_ incorporation. Electron localization function maps revealed enhanced electron density near the Cr_2_O_3_–NiFeOOH interface, contrasting with the uniform charge distribution in unmodified NiFeOOH (Fig. S2) [16]. These results demonstrate that Cr_2_O_3_ introduction facilitates interfacial electron transfer, establishing an electronic foundation for efficient OER catalysis (Fig. 1a and b, insets).

**Figure 2.**
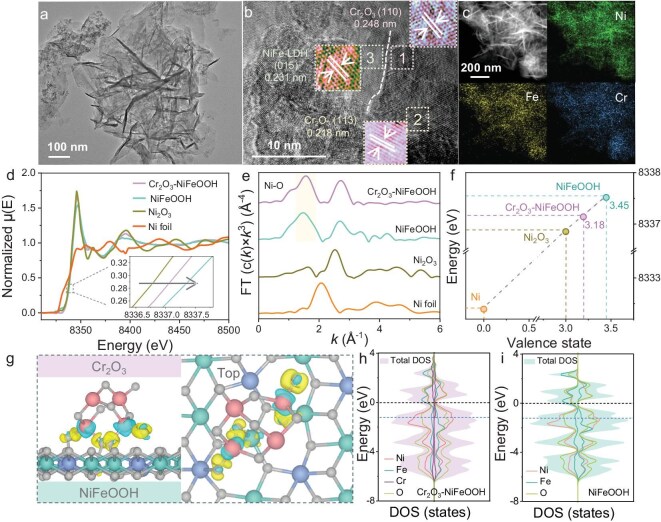
Characterization of Cr_2_O_3_–NiFeOOH nanosheets. (a) TEM image, (b) high-resolution TEM image and (c) corresponding elemental distributions of Cr_2_O_3_–NiFe-LDH. (d) Ni K-edge X-ray absorption near-edge structure (XANES) spectra and (e) Fourier transform of extended X-ray absorption fine structure (FT-EXAFS). (f) XANES valence-state fitting line graph. (g) Differential charge density diagram of Cr_2_O_3_–NiFeOOH. Density of states (DOS) of (h) Cr_2_O_3_–NiFeOOH and (i) NiFeOOH.

Cr_2_O_3_–NiFe-LDH was obtained by using hydrothermal and electrodeposition in two steps. The 3D architecture of conductive nickel foam (NF) offers an extensive and robust interface for catalyst growth (Fig. S3). Scanning electron microscopy images (Fig. S4) and the transmission electron microscopy (TEM) image (Fig. 2a) reveal the nanosheet array structure of Cr_2_O_3_–NiFe-LDH, which shows no significant morphological difference compared with the nanosheet structure of NiFe-LDH (Fig. S5). The crystal structure of Cr_2_O_3_–NiFe-LDH was analysed by using X-ray diffraction (XRD), showing characteristic diffraction peaks corresponding to NiFe-LDH (PDF#40-0215) along with weak characteristic peaks attributed to Cr_2_O_3_ (PDF#38-1479), which verify the coexistence of Cr_2_O_3_ and NiFe-LDH in the synthesized catalyst (Fig. S6). High-resolution TEM analysis revealed the presence of Cr_2_O_3_ nanoparticles with lattice fringes of 0.218 and 0.248 nm corresponding to the (113) and (110) planes, and there exists a clear interface structure between the Cr_2_O_3_ and NiFe-LDH (Fig. 2b). Additionally, elemental mapping via TEM-energy dispersive spectroscopy confirmed the homogeneous distribution of Cr throughout the material (Fig. 2c). The surface of the catalysts inevitably undergoes phase reconfiguration during the OER process to form oxyhydroxide, denoted as (Cr_2_O_3_–NiFeOOH), resulting in a change in the active substance of the bulk catalyst. The Raman spectra of Cr_2_O_3_–NiFeOOH were analysed and the peaks at 308.9 and 563.4 cm^−1^ indicate the formation of Cr_2_O_3_ (Fig. S7) [17]. Specifically, Fig. S8 shows the XRD patterns after the reaction of NiFe-LDH with and without Cr_2_O_3_ loading. The diffraction peak near 23° corresponding to the (006) crystal plane shows almost no angular shift, indicating that Cr_2_O_3_ is only loaded on the NiFeOOH surface [18]. X-ray photoelectron spectroscopy (XPS) revealed a positive shift in the Ni 2p orbitals of Cr_2_O_3_–NiFeOOH, accompanied by an increase in the Ni^3+^/Ni^2+^ ratio from 19% to 24% (Figs S9 and S10), highlighting the role of Cr in improving the catalytically active Ni^3+^ state [19]. X-ray absorption near-edge structure (XANES) analysis revealed a distinct positive shift in the spectra of Cr_2_O_3_–NiFeOOH and NiFeOOH relative to NiO and Ni_2_O_3_ (Fig. 2d), providing direct evidence of the formation of the catalytically active NiOOH phase during the OER-induced phase transition. The extended Ni–O coordination bond in Cr_2_O_3_–NiFeOOH indicates a relatively weaker binding affinity between Ni and O, which protects it from overoxidation during the OER and thereby preserves its catalytic activity (Fig. 2e). Quantitative analysis of the average Ni valence states showed values of approximately 3.18 and 3.45 for Cr_2_O_3_–NiFeOOH and NiFeOOH, respectively (Fig. 2f). Further corroboration was provided by wavelet transform (WT) analysis of the EXAFS data, which confirmed that the local coordination environment of Cr_2_O_3_–NiFeOOH and NiFeOOH is highly similar to that of NiOOH and Ni_2_O_3_ (Fig. S11). The differential charge density diagram in Fig. 2g reflects a strong interaction between Cr_2_O_3_ and NiFeOOH, with Cr surface electrons transferred to the NiFeOOH surface via Cr–O–Ni/Fe [20]. As can be seen from the DOS, Cr_2_O_3_–NiFeOOH exhibits a state density distribution similar to that of NiFeOOH. However, near the Fermi level, Cr_2_O_3_–NiFeOOH shows a higher number of energy bands, indicating that the surface of Cr_2_O_3_–NiFeOOH has a stronger electron-transfer capacity, which is conducive to the electron transfer between the catalyst and the reactants in the reaction (Fig. 2h and i).

### Theoretical investigations of Cr–O–Ni asymmetric bonds

**Figure 3.**
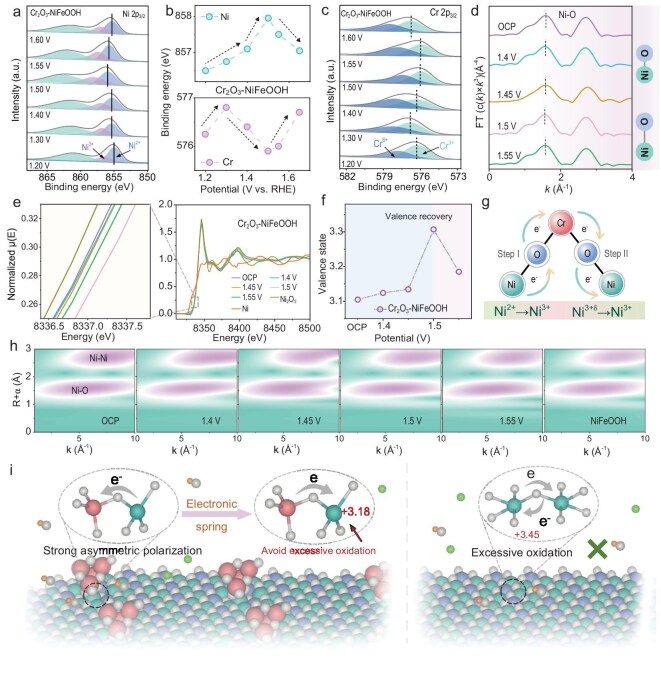
XPS spectra of (a) Ni 2p and (c) Cr 2p for Cr_2_O_3_–NiFeOOH under different potentials. (b) Bond energy offset of Ni and Cr according to (a) and (c). (d) Fourier transform of EXAFS spectra for Cr_2_O_3_–NiFeOOH under different potentials. (e) XANES spectra of Cr_2_O_3_–NiFeOOH under different potentials. (f) Valence state of Ni varies with the different potentials. (g) Electron-transfer diagram of Cr–O–Ni bond. (h) WT plots of Cr_2_O_3_–NiFeOOH under different potentials. (i) Schematic diagram of dynamic regulation of asymmetric active bonds.

The valence-state evolution of Ni and Cr in Cr_2_O_3_–NiFeOOH during the transition from the non-Faradaic region to the OER region (1.20–1.60 V) was investigated by using XPS to elucidate the elastic regulation of electrode valence states via the Cr–O–Ni bridging oxygen bond during the OER. As shown in Fig. 3a–c, at the pre-catalytic stage (<1.23 V), the binding energies of both Cr and Ni increase slightly. However, upon initiation of the OER, Cr and Ni display opposite trends with increasing potential, where Cr shifts to lower binding energies and Ni shifts to higher binding energies. The hypervalent Ni formed under these conditions serves as the true active site for the OER, driving the subsequent catalytic process. Notably, as the voltage increases further, the valence state of Cr is restored after 1.5 V, while Ni gains electrons to prevent overoxidation and deactivation. Through analysis of the XPS spectra at different potentials, a quantitative assessment of the charge transfer in Cr_2_O_3_–NiFeOOH was conducted. Before 1.5 V, the electron loss from Ni amounts to ∼1.85 eV, whereas, after 1.5 V, the dynamic modulation from Cr reduces the electron loss of Ni to 0.55 eV. In contrast, the Ni 2p orbitals in NiFeOOH are continuously oxidized throughout the OER, shifting toward higher binding energies (Fig. S12). These findings suggest that the Cr–O–Ni bridging oxygen bond in Cr_2_O_3_–NiFeOOH functions as an ‘electron spring’ during the OER process, dynamically regulating the valence states of the electrode elements and facilitating the reaction progression. To further investigate the mechanism behind the electron oscillations observed in the OER process mediated by the Cr–O–Ni bond, X-ray absorption spectrum (XAS) measurements of the Ni K-edge were conducted under varying oxidation potentials [21]. Careful analysis of the Fourier transform of extended X-ray absorption fine structure (FT-EXAFS) data (Fig. 3d) reveals that, between 1.45 and 1.50 V, the shortening of the Ni–O bond length in the first coordination shell is attributed to the enhanced coordination structure around Ni due to its high oxidation state at the metal center. Beyond 1.50 V, reverse electronic regulation by Cr reduces the Ni–O coordination, as evidenced by the increased bond length, indicating a decrease in the oxidation state of Ni [22]. Additionally, the Ni K-edge XANES spectrum shows a positive shift in the near-edge absorption edge with increasing OER potential, confirming that the average oxidation state of Ni increases during the OER process (Fig. 3e). An inflection point is observed at 1.50 V, where the oxidation state of Ni stabilizes at +3.18, effectively preventing an uncontrolled rise in oxidation (Fig. 3f). This observation is consistent with the quasi *in situ* XPS results, highlighting the critical role of the Cr–O–Ni bond in maintaining the catalytic stability and efficiency of Ni during the OER. These results conclusively demonstrate that the incorporation of Cr facilitates the formation of Cr–O–Ni asymmetric bridging oxygen bonds, effectively suppressing the overoxidation of Ni and stabilizing it in its most catalytically active state (+3), thereby enhancing the catalytic performance (Fig. 3g). The Ni–O coordination environment of the WT analysis further confirms the results of the EXAFS (Fig. 3h) [19]. Based on the above analysis, a novel OER mechanism mediated by the dynamic Cr–O–Ni asymmetric electron-bridging bond is proposed. As illustrated in Fig. 3i, the bridging oxygen acts as a dynamic ‘electronic spring’ throughout the OER process. Initially, due to asymmetric polarization, electrons are transferred from Ni to Cr, facilitating the formation of hypervalent Ni species as the active catalytic site. Beyond 1.50 V, electron back-donation from Cr to Ni prevents the overoxidation of Ni, thereby ensuring that the catalyst remains in a highly active state. Concurrently, the oxidation state of Cr increases due to electron depletion, enhancing its ability to strongly adsorb OH^−^. This synergistic interplay between the hyperactive Ni species and the OH^−^ accumulation on the Lewis acid of the Cr sites collectively drives the efficient catalysis of the alkaline OER in seawater.

### Investigation of reaction mechanism

**Figure 4.**
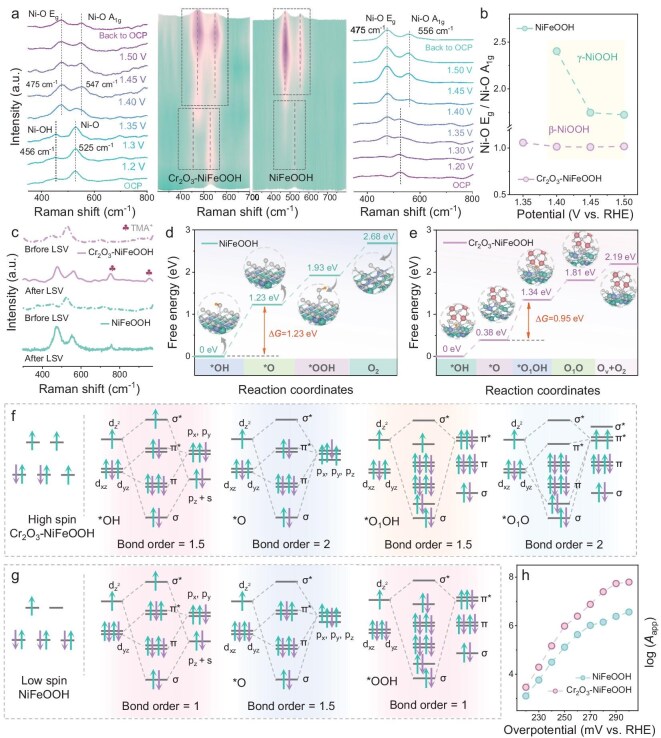
(a) *In situ* Raman spectra of Cr_2_O_3_–NiFeOOH and NiFe-LDH in 1.0 M KOH + seawater. (b) Peak area ratio based on (a). (c) Raman spectra before and after operating in 1.0 M tetramethylammonium hydroxide (TMAOH) solution. Free-energy diagrams of AEM on (d) NiFeOOH and lattice oxygen-mediated mechanism on (e) Cr_2_O_3_–NiFeOOH, respectively. Schematic illustration of orbital hybridization between Ni^3+^ and reaction intermediates under (f) high spin and (g) low spin. (h) Logarithm of pre-exponential factor *A*_app_ for catalysts at fixed overpotentials.

The mechanism by which Cr_2_O_3_ incorporation enhances the activity of NiFeOOH was investigated by using *operando* Raman spectroscopy. As shown in Fig. 4a, with increasing applied potential, the characteristic *γ*-NiOOH phase emerges on the Cr_2_O_3_–NiFeOOH surface, as evidenced by the Raman peak shift from 456/525 to 475/547 cm^−1^; however, this structural transition occurs at significantly higher potentials for NiFeOOH electrodes [19,23]. Both *γ*-NiOOH and *β*-NiOOH exhibit a pair of characteristic bands at approximately 475 and 547 cm^−1^, with distinct differences in their relative intensities. Specifically, *γ*-NiOOH demonstrates a higher vibrational intensity ratio (*E*_g_/*A*_1g_) between the 475- and 547-cm^−1^ bands compared with *β*-NiOOH (Fig. 4b) [24]. Thus, the *E*_g_/*A*_1__g_ ratio serves as a reliable indicator of the phase composition and structural disorder within the NiOOH matrix [25]. It is widely recognized that *β*-NiOOH is the more catalytically active phase for the OER compared with its *γ*-counterpart. However, *β*-NiOOH tends to undergo a topochemical transformation into the less active *γ*-NiOOH, making it crucial to prevent the overoxidation of *β*-NiOOH during the OER [26]. The observed higher *E*_g_/*A*_1__g_ value for Cr_2_O_3_–NiFeOOH compared with NiFeOOH suggests that the incorporation of Cr_2_O_3_ effectively suppresses the overoxidation of NiFe-LDH, thereby stabilizing the more active *β*-NiOOH phase and enhancing the catalytic performance [27]. The kinetics of Cr_2_O_3_–NiFeOOH in the seawater electrolysis OER were studied intensively. Operando electrochemical impedance spectroscopy (EIS) serves as a powerful tool for probing the electrode/electrolyte interface properties and the adsorption kinetics of reactants on the electrode surface. As shown in Fig. S13, the Nyquist plots of Cr_2_O_3_–NiFeOOH and NiFeOOH in 1.0 M KOH + seawater within a potential range of 1.11–1.47 V demonstrate that Cr_2_O_3_–NiFeOOH exhibits significantly lower charge-transfer resistance across all applied potentials. This reduction highlights the role of the Cr–O–Ni bridging bonds in accelerating the interfacial charge transfer, thereby enhancing the surface activation of the electrocatalyst. Additionally, Bode plots recorded at various potentials provide further insights into the electrochemical processes underlying the OER catalysis. These plots reveal distinct surface-reaction processes occurring in specific frequency response intervals: (i) the adsorption capacitance in the high-frequency region (∼10^4^ Hz), attributed to the rapid relaxation processes, is associated with the adsorption of oxygen species (e.g. OH^−^); (ii) the Faradaic process in the intermediate-frequency range (∼10^2^ Hz) is linked to charge-transfer impedance; and (iii) double-layer capacitance in the low-frequency region (<10 Hz) is governed by diffusion processes [28]. As shown in Fig. S14, both Cr_2_O_3_–NiFeOOH and NiFeOOH exhibit pronounced Faradaic processes in the intermediate-frequency region, confirming that faster interfacial electron transfer contributes to improved OER performance. These findings underscore the role of the enhanced charge-transfer kinetics facilitated by Cr–O–Ni interactions in promoting efficient oxygen-evolution catalysis. Electrochemical reactions are typically classified into two categories: proton-coupled electron transfer (PET, which usually represents a lattice oxygen-mediated mechanism (LOM) path) and concerted proton-coupled electron transfer (*c*-PET, which usually represents an AEM path), depending on whether the proton and electron transfers occur simultaneously or sequentially [29]. The overlap of the proton vibrational wave functions plays a critical role in determining the OER kinetics and kinetic isotope effects (KIEs). for the c-PET reaction, as it is highly sensitive to the proton donor–acceptor separation [30]. When this distance shortens, the enhanced wave function overlap accelerates the reaction rate while suppressing KIEs [31]. Notably, Cr_2_O_3_–NiFeOOH demonstrates lower KIEs compared with NiFeOOH alone (Fig. S15), indicating that Cr_2_O_3_ near the active sites promotes more efficient OER electrocatalysis. Further, tetramethylammonium cation (TMA^+^) was used as a chemical probe to reconfirm the surface-reaction path. After the replacement of K^+^ with TMA^+^ in alkaline solution, the activity change in Cr_2_O_3_–NiFeOOH is significantly greater than that in NiFeOOH (Fig. S16), which indicates that TMA^+^ on Cr_2_O_3_–NiFeOOH has a stronger electrostatic reaction with oxidation intermediates [17]. Consequently, this interaction inhibits lattice oxygen-mediated O_2_ evolution on Cr_2_O_3_–NiFeOOH, leading to a reduced current density. Raman spectroscopy, as shown in Fig. 4c, was used to record the characteristics of TMA^+^ on different catalysts. The LOM pathway is hindered by strong binding between the TMA^+^ and Cr_2_O_3_–NiFeOOH electronegative oxygen species produced during the OER, thus explaining the decrease in activity, which confirms that the incorporation of Cr transforms the AEM mechanism of NiFeOOH into an LOM mechanism [32]. Additionally, *in situ* attenuated total reflection surface-enhanced infrared absorption spectroscopy was employed to investigate the interaction between the oxygen-containing intermediates and the catalytic surface during the OER. As shown in Fig. S17, two distinct peaks at approximately 1018 and 1174 cm^−1^ are observed for NiFeOOH, whose intensities gradually increase with the applied anodic potential [8,33–35]. These peaks can be assigned to the *OOH intermediate generated via the AEM. In contrast, Cr_2_O_3_–NiFeOOH exhibits more prominent stretching vibration peaks of the characteristic *OO intermediate from the LOM at 1060, 1220 and 1533 cm^−1^ [34–36]. These spectroscopic pieces of evidence collectively demonstrate that Cr_2_O_3_–NiFeOOH and NiFeOOH follow the LOM and AEM pathways, respectively. Figure 4d and e explain the evolution of surface oxygen in the AEM/LOM path based on DFT calculations. For Cr_2_O_3_–NiFeOOH, the energy barrier from *O to *O_1_OH is higher than those of other steps, indicating that this step is a rate-determining step (RDS) [37]. The RDS of NiFeOOH is the adsorption of OH^−^. The adsorption energy of Cr_2_O_3_–NiFeOOH@NF for RDS is only 0.58 eV, which is lower than that of NiFeOOH@NF (2.23 eV), owing to the strong selectivity of Cr_2_O_3_ for OH^−^ and the formation of more active Ni species. Figure 4f and g illustrate the distinct coupling configurations between the d-orbitals and the adsorbed intermediate orbitals. The spin states of the catalysts (Cr_2_O_3_–NiFeOOH and NiFeOOH, corresponding to high-spin and low-spin Ni^3+^, respectively, as shown in Fig. S18) were qualitatively evaluated through zero-field-cooled temperature-dependent magnetic susceptibility (*χ*) measurements. Bond-order analysis of the intermediates interacting with the two spin-state configurations reveals that a higher bond order corresponds to stronger adsorption capacity. For *OH adsorption, the bond order is 1 on low-spin Ni^3+^ sites, whereas it increases to 1.5 on high-spin Ni^3+^ sites in Cr_2_O_3_–NiFeOOH. This enhanced adsorption strength suggests that high-spin Ni^3+^ active sites facilitate the initial step of the OER cycle by promoting *OH binding. Temperature-dependent kinetic analysis was performed to investigate the apparent pre-exponential factor (*A*_app_) of Cr_2_O_3_–NiFeOOH@NF and NiFeOOH@NF catalysts during the alkaline OER. Linear sweep voltammetry (LSV) measurements were conducted for both catalysts in 1.0 M KOH + seawater solution over a temperature range of 25°C–65°C (Figs S19 and S20). As expected, the catalytic performance of both catalysts improved with increasing temperature. The *A*_app_ values at fixed overpotentials were determined by using the Arrhenius equation [29]. The observed difference in *A*_app_ between the two catalysts suggests that the incorporation of Cr_2_O_3_ alters the activation entropy, which is associated with the number of active intermediates participating in the RDS, as well as the interfacial concentration of the active sites [38]. These changes contribute to the enhanced intrinsic activity of the catalytic active sites in Cr_2_O_3_–NiFeOOH (Fig. 4h).

### OH^−^ selective adsorption

**Figure 5.**
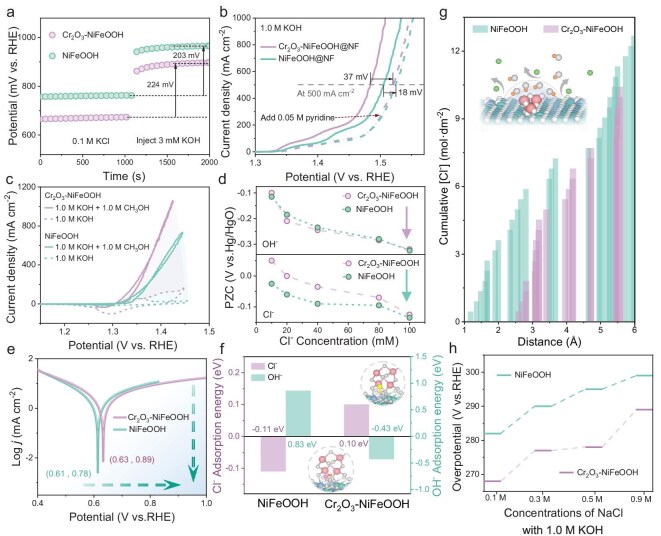
(a) OCP measured in 0.1 M KCl solution before and after the injection of 3 mM of KOH over Cr_2_O_3_–NiFeOOH and NiFeOOH. (b) Current densities of Cr_2_O_3_–NiFeOOH and NiFeOOH in 1.0 M KOH before and after the addition of 0.05 M pyridine. (c) Cyclic voltammetry plots in 1.0 M KOH with and without 1.0 M methanol for Cr_2_O_3_–NiFeOOH and NiFeOOH. (d) Potentials of zero charge of Cr_2_O_3_–NiFeOOH and NiFeOOH electrodes in electrolytes with different concentrations of KOH and NaCl. (e) Tafel plots. (f) Adsorption energies of Cl^−^ and OH^−^ for Cr_2_O_3_–NiFeOOH and NiFeOOH. (g) Profiles of cumulative Cl^−^ concentration as a function of position (distance) perpendicular to different samples. (h) Step diagram of Cl^−^-concentration gradient.

The adsorption capacity of the electrode surface was evaluated by using the open-circuit potential (OCP) method. Upon the addition of 3 mM KOH, the OCP of Cr_2_O_3_–NiFeOOH exhibits a larger shift (224 mV) compared with NiFeOOH (203 mV), indicating stronger OH^−^ adsorption on the surface of Cr_2_O_3_–NiFeOOH@NF (Fig. 5a) [39,40]. To further investigate the microenvironment at the anode–electrolyte interface, with a particular focus on the role of Cr_2_O_3_ components in the catalyst, pyridine, a Lewis base, was employed as a probe molecule. According to Lewis-acid–base theory, pyridine preferentially adsorbs on Lewis-acid sites, thereby inhibiting OH^−^ adsorption on the catalyst surface [39]. As shown in Fig. 5b, after the addition of 0.05 M pyridine, the current density of Cr_2_O_3_–NiFeOOH decreased by approximately twice that of NiFeOOH at 1.4 V (vs. reversible hydrogen electrode (RHE)). This result highlights the role of high-valence-state Cr as a strong Lewis acid, effectively promoting OH^−^ adsorption and enhancing catalytic performance. The electrophilic nature of OH* species generated during the OER can be evaluated by their interaction with nucleophilic alcohols (e.g. methanol, ethanol or isopropanol) under typical OER conditions. Cyclic voltammetry measurements were carried out for NiFeOOH and Cr_2_O_3_–NiFeOOH electrodes in 1.0 M KOH solution, with and without the addition of methanol. As shown in Fig. 5c, the shaded areas represent the current differences arising from the methanol-oxidation reaction, where the area is proportional to the charge transfer, reflecting the degree of the reaction [41]. A significantly larger shaded area was observed for Cr_2_O_3_–NiFeOOH compared with NiFeOOH, demonstrating that the incorporation of Cr_2_O_3_ improves the stabilization of and interaction with OH* intermediates, thereby enhancing the catalytic activity during the OER. The enhanced OH^−^ adsorption, attributed to the ion-shielding effect, creates a protective layer that effectively blocks the penetration of corrosive Cl^−^ ions in the seawater, thereby facilitating the efficient and stable OER catalysis of the Cr_2_O_3_–NiFeOOH electrode in alkaline seawater. The potential of zero charge (PZC) is defined as the potential at which no excess charge exists on the electrode surface. It is determined from the minimum of the differential capacitance curve in a dilute electrolyte and serves as a direct indicator for evaluating specific adsorption behavior and adsorption capacity on the electrode surface [39]. As shown in Fig. 5d and Figs S21–S25, the PZCs of both Cr_2_O_3_–NiFeOOH and NiFeOOH shift negatively with increasing concentrations of OH^−^ and Cl^−^, indicating competitive adsorption between these ions. Upon the addition of OH^−^, the PZC of Cr_2_O_3_–NiFeOOH exhibits a more pronounced negative shift compared with NiFeOOH, suggesting stronger OH^−^ adsorption on the Cr_2_O_3_–NiFeOOH surface. Conversely, after the addition of Cl^−^, the negative shift in the PZC of Cr_2_O_3_–NiFeOOH is less significant than that of NiFeOOH, confirming that Cr_2_O_3_–NiFeOOH possesses stronger OH^−^-adsorption and superior Cl^−^-shielding capabilities. These observations align with the Esin–Markov effect, further highlighting the enhanced surface properties of Cr_2_O_3_–NiFeOOH. As exhibited in Fig. 5e, the corrosion potential (*E*_corr_) of the Cr_2_O_3_–NiFeOOH electrode increases compared with that of NiFeOOH, further indicating that the catalyst's corrosion resistance to seawater is improved by the introduction of Cr_2_O_3_ [4,42]. Furthermore, DFT calculations were employed to verify the selective adsorption of Cl^−^ and OH^−^ ions by high-valence Cr species on the electrode surface and their role in enhancing the anti-corrosion properties during seawater splitting. As shown in Fig. 5f, the Gibbs adsorption energy for Cl^−^ on Cr_2_O_3_–NiFeOOH (0.10 eV) is significantly higher than that on pure NiFeOOH (−0.11 eV), indicating that high-valence Cr ions effectively inhibit Cl^−^ adsorption while selectively adsorbing OH^−^ [43]. This selective adsorption prevents Cl^−^ corrosion and suppresses the undesirable ClOR. To further validate the Cl^−^ resistance mechanism of the NiFeOOH electrode during seawater splitting following the deposition of Cr_2_O_3_ on its surface, classical molecular dynamics simulations were performed (Fig. 5g). The simulations depict the total Cl^−^ concentration in the electrolyte as a function of the distance from the electrode surface. The cumulative concentration, representing the integrated concentration within a given distance from the surface, reveals that the presence of Cr_2_O_3_ on the electrode increases the average distance of Cl^−^ from the surface (∼1.41 Å) [44]. This behavior is attributed to the enrichment of OH^−^ on the electrode surface, which induces the electrostatic repulsion of Cl^−^. As shown in Fig. 5h and Fig. S26, the presence of high Cl^−^ concentrations in the NaCl + KOH solutions leads to a noticeable reduction in the OER catalytic performance of both Cr_2_O_3_–NiFeOOH and NiFeOOH compared with their performance in pure 1.0 M KOH [45]. However, Cr_2_O_3_–NiFeOOH demonstrates significantly less performance degradation under these conditions, confirming that the incorporation of Cr_2_O_3_ species enhances the corrosion resistance of NiFeOOH, particularly in environments with high Cl^−^ concentrations.

### Performance of AEM seawater electrolyser

**Figure 6.**
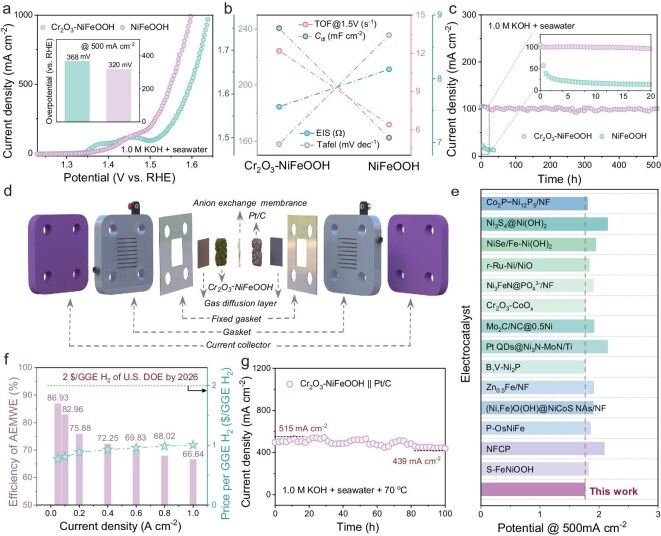
(a) Polarization curves of Cr_2_O_3_–NiFeOOH and NiFeOOH for the OER in different solutions. (b) Some major OER performance metrics of Cr_2_O_3_–NiFeOOH and NiFeOOH in 1.0 M KOH + seawater. (c) Durability test of the OER at a current density of 0.1 A cm^−2^ in 1.0 M KOH + seawater electrolyte. (d) Schematic diagram of the AEM electrolyser. (e) Comparison of Cr_2_O_3_–NiFeOOH with other reported materials in AEM electrolyser with alkaline seawater. (f) Efficiency and price of per GGE H_2_ of AEM electrolyser. (g) Stability of AEM electrolyser operating at 0.5 A cm^−2^ in 1.0 M KOH + seawater electrolyte.

The OER activity of the catalysts was first systematically evaluated in 1.0 M KOH. As shown in Fig. S27, the overpotential of Cr_2_O_3_–NiFeOOH required to achieve a current density of 1.0 A cm^−2^ is as low as 277 mV, significantly outperforming NiFeOOH (304 mV) and even surpassing that of the commercial RuO_2_. Tafel slope analysis was conducted to assess the catalytic kinetics of the RDS. The decreased Tafel slope from 126.7 to 120.9 mV dec^−1^ indicates the promoted OER kinetics with the incorporation of Cr_2_O_3_. EIS measurements further confirm that Cr_2_O_3_ doping reduces the charge-transfer resistance (*R*_ct_) of the Cr_2_O_3_–NiFeOOH electrode (Fig. S28) [46]. Furthermore, the electric double-layer capacitance (*C*_dl_) of NiFeOOH increases from 1.97 to 2.96 mF cm^−2^ after coupling with Cr_2_O_3_ (Fig. S29), reflecting an expansion of the electrochemically active surface area. Additionally, the turnover frequency of Cr_2_O_3_–NiFeOOH is determined to be 13.65 s^−1^ at an overpotential of 1.45 V (Fig. S30), which is ∼1.09 times that of NiFeOOH, underscoring the significantly enhanced intrinsic catalytic activity for the OER resulting from the incorporation of Cr_2_O_3_. As depicted in the radar diagram in Fig. S31 and Table S1, Cr_2_O_3_–NiFeOOH demonstrates superior overall OER performance in alkaline solutions. Its low overpotential for delivering high current densities in 1.0 M KOH highlights its potential for large-current-density seawater oxidation without initiating the ClOR process [47]. Specifically, Cr_2_O_3_–NiFeOOH exhibits a smaller potential increase at 500 mA cm^−2^ (320 mV) when transitioning from alkaline pure water to an alkaline-seawater electrolyte compared with NiFeOOH (368 mV), indicating that the incorporation of Cr_2_O_3_ significantly improves both the catalytic activity and the corrosion resistance (Fig. 6a). Furthermore, the other electrocatalytic indicators of Cr_2_O_3_–NiFeOOH are all superior to those of NiFeOOH in the alkaline-seawater system (Figs S32–S34, Fig. 6b and Table S2). Under a high current density of 100 mA cm^−2^, the Cr_2_O_3_–NiFeOOH electrode exhibits exceptional durability in a 1.0 M KOH + seawater electrolyte, maintaining stable operation for >500 h with a low decrease in the current and the morphology of the catalyst is maintained after stabilization. In contrast, the current of NiFeOOH drops rapidly by ∼80 mA cm^−2^ within 20 h under the same conditions and the nanosheet structure undergoes partial curling and agglomeration, which reduces the active surface area and consequently diminishes its catalytic activity (Fig. 6c and Fig. S35). Chemical state analysis was performed on the sample surface after stability testing. The Ni 2p and Fe 2p orbitals in Cr_2_O_3_–NiFeOOH exhibit a shift toward lower binding energies compared with those in pristine NiFeOOH, which aligns with the previously mentioned role of Cr_2_O_3_ in preventing overoxidation. Moreover, it was observed that the Cr element still exists (Fig. S36). Furthermore, the Cr-containing electrode achieves higher Faradaic efficiency for O_2_ production than NiFeOOH, with excellent four-electron OER selectivity facilitated by the Cr_2_O_3_ species (Figs S37–S40). The Faraday efficiency slightly exceeding 100% also confirmed that the lattice oxygen in Cr_2_O_3_–NiFeOOH participated during the OER reaction. The formation of hypochlorite in the electrolyte was further investigated by using a colorimetric method. Free chlorine species (Cl_2_, hypochlorite and chlorite) oxidize *N,N*-diethyl-*p*-phenylenediamine to generate a pink dye, the strong absorption of which was quantified via ultraviolet-visible (UV–vis) spectrophotometry. As expected, only trace amounts of hypochlorite were detected in the electrolyte after the seawater electrolysis, further corroborating the exceptional ability of the Cr_2_O_3_–NiFeOOH catalyst to suppress the ClOR and maintain high OER selectivity under seawater-splitting conditions (Figs S41 and S42). Inspired by the excellent OER properties of the electrodes in alkaline seawater, Cr_2_O_3_–NiFeOOH as the anode was further assembled into a closely packed anion-exchange-membrane electrolytic cell (AEMEC) (Fig. 6d and Fig. S43). As shown in Fig. S44, under LSV testing conditions, the Cr_2_O_3_–NiFeOOH||Pt/C electrolytic cell achieves a current density of 1.0 A cm^−2^ at a cell voltage of only 1.88 V, which is superior to those of most recently reported AEM electrolysers in alkaline seawater (Fig. 6e and Table S3). Furthermore, the cell efficiency of the Cr_2_O_3_–NiFeOOH||Pt/C electrolyser reaches an impressive 82.96% at a current density of 100 mA cm^−2^ (Fig. 6f), significantly exceeding the 2020 US Department of Energy (DOE) target of 77%, with sustained high efficiency even at ampere-level current densities [48]. Economic analysis based on Fig. 6f and Table S4 indicates that the hydrogen-production cost at a current density of 1.0 A cm^−2^ is as low as $0.87 per GGE H_2_, which is less than half the US DOE’s 2026 target of $2.00 per GGE H_2_ [49]. This demonstrates the cost-effectiveness and scalability of the Cr_2_O_3_–NiFeOOH catalyst in practical applications. The durability of the Cr_2_O_3_–NiFeOOH||Pt/C electrolytic cell was further evaluated. The cell operated continuously for >100 h at a voltage of 1.78 V, with a minimal current density degradation of only 76 mA cm^−2^ over the testing period (Fig. 6g). The slight fluctuations observed in the data are likely due to gas accumulation at high current densities. These results highlight the outstanding catalytic stability, high efficiency and cost-effectiveness of Cr_2_O_3_–NiFeOOH electrodes, confirming their potential for industrial-scale AEM seawater electrolysis.

## CONCLUSION

In summary, inspired by the concept of a spring, a Cr–O–Ni asymmetric reactive oxygen bridging catalyst of Cr_2_O_3_–NiFe-LDH has been developed, in which the bridging oxygen functions as a dynamic ‘electronic spring’ during the OER process. *In situ* Raman spectroscopy, quasi *in situ* XPS and *in situ* Fourier transform of the EXAFS spectra confirm that the Cr–O–Ni bond facilitates the phase transition of Ni to its active state at low potentials and the mechanism transition from AEM to LOM in the oxidation process of Cr_2_O_3_–NiFeOOH, thereby reducing the RDS barrier. Alternatively, at high potentials, the electron transfer from Cr to Ni prevents the over-transformation of the active substance NiFeOOH from the active *β*-phase to the *γ*-phase, stabilizing Ni at a +3 active valence state. The reverse electronic regulation of Cr reduces Ni–O coordination and elongates the bond length, effectively preventing the deactivation caused by uncontrolled Ni oxidation. Furthermore, the high-valence Cr enhances the adsorption and mass transfer of OH^−^ on the anode surface, suppressing Cl^−^ adsorption and improving both the activity and the stability (over 500 h) of the OER in alkaline seawater. Utilizing the Cr_2_O_3_–NiFeOOH||Pt/C catalyst in an AEM electrolyser achieves a current density of 1.0 A cm^−2^ at a cell voltage of only 1.88 V and an energy-conversion efficiency of 77.9% at 100 mA cm^−2^, with a hydrogen-production cost of $0.85 per GGE, which is significantly lower than the 2026 DOE target of $2.00/GGE. These results provide a promising strategy for the development of highly active and stable electrocatalysts for seawater electrolysis and OER applications.

## Supplementary Material

nwag091_Supplemental_File
