# Supplementary material for: Design of asymmetric electronic spring for stabilizing selective seawater oxidation
Source: Natl Sci Rev. 2026 Feb 10;13(8):nwag091. doi: 10.1093/nsr/nwag091 (PMC13156940; doi:10.1093/nsr/nwag091)
Supplement: nwag091_Supplemental_File [file nwag091_supplemental_file.pdf]

Design of asymmetric electronic spring for stabilizing selective seawater oxidation

Lili Guo[1,2], Chao Feng[3], Jingqi Chi[1,*], Tianrong Zhan[4], Zekun Wang[4], Hailing Guo[2], Zhi Su[5,*], Xiaobin Liu[1,*], Zexing Wu[1,4], Jianping Lai[1,4], Lei Wang[1,4,*]

[1]Key Laboratory of Eco-chemical Engineering, International Science and Technology Cooperation Base of Eco-chemical Engineering and Green Manufacturing, College of Chemical Engineering, Qingdao University of Science and Technology, Qingdao 266042, PR China.
[2]State Key Laboratory of Heavy Oil Processing, College of Chemical Engineering, China University of Petroleum (East China), Qingdao 266580, China.
[3]College of Chemical and Biological Engineering, Shandong University of Science and Technology, Qingdao, Shandong 266590, China
[4]College of Chemistry and Molecular Engineering, Qingdao University of Science and Technology, Qingdao 266042, PR China.
[5]State Key Laboratory of Chemistry and Utilization of Carbon, Based Energy Resources, College of Chemistry, Xinjiang University, Urumqi, 830017, Xinjiang, PR China

E-mail: chijingqi@qust.edu.cn; suzhixj@sina.com; liuxb@qust.edu.cn; inorchemwl@126.com

Experimental Procedures

Material Synthesis. For NiFe LDH, First, First, 2.0 cm × 3.0 cm Ni foam (NF) was sonicated for 20 min in 0.1 M hydrochloric acid solution, acetone, and ethanol, respectively to to remove the oxides and organic species on the surface and obtain the cleaned NF. Then, $Ni(NO_3)_2 \cdot 6H_2O$ (0.2908 g), $Fe(NO_3)_3 \cdot 9H_2O$ (0.2020 g), and urea (0.3003 g) were dissolved in 30 mL DI water under ultrasonic treatment for 30 min. Afterward, the resultant solution was transferred to a 50 mL Teflon autoclave for a 12 h hydrothermal reaction at 120 ^{o}C. After the hydrothermal reaction is completed, NF was washed with deionized water and ethanol and dried in the oven to obtain the NiFe-LDH. Cr_2O_3-NiFe-LDH was synthesized by an electrodeposition method. Typically, 0.120 g $Cr(NO_3)_3 \cdot 9H_2O$ were dissolved in 50 mL DI water as electrolyte. The prepared NiFe-LDH was employed as working electrode, and the Hg/HgO, Pt wire were used as reference and counter electrodes, respectively. The electrodeposition was conducted by a cathodic voltage of -1 V for 900 s.

Electrochemical Measurements. The electrochemical measurements of all samples were performed on a Gamry Reference 3000 workstation using a standard three-electrode system, and the prepared 1.0 cm × 1.0 cm sample, Hg/HgO (0.098V vs. RHE) electrode and graphite rod were employed as the working electrode, reference electrode and counter electrode, respectively. All the potentials applied in this experiment were calibrated to the reversible hydrogen electrode (RHE) using the equation: (RHE): $E_{RHE} = E_{Hg/HgO} + 0.0592pH + 0.098$. The catalytic activities were evaluated in 1.0 M KOH and 1.0 M KOH+seawater solutions, the natural seawater from Stone old man bathing beach, Qingdao, China. The linear sweep voltammetry (LSV) curves were recorded at a scan rate of 10 mV s^{-1} with iR compensation. The Tafel plots were constructed by η=a+b log j, (η is the overpotential, a is content, b is the Tafel slope, and j is the current density). The electrochemical impedance spectroscopy (EIS) measurements were recorded in the frequency range from 0.1 Hz to 100 kHz with amplitude of 5 mV. The stability of Cr_2O_3-NiFeOOH were tested by chronoamperometry i-t measureme

nts in different solutions at room temperature or 60 ^{o}C. The absolute voltammetric charges can be obtained from the CVs ranging from -0.2 V to 0.6 V (vs. RHE) in pH=7 phosphate buffer solution. The calculations of n and TOF can be determined in the following equations:

$$n = Q/2F$$

$$TOF = I/2nF$$

(Q is the number of voltammetric charges (C); I is current (A) during the linear sweep measurement; F is Faraday constant (96500 C mol^{-1}); n is the number of active sites (mol). The factor of 1/2 is 1/2 for HER and 1/4 for OER.)

To extract the double-layer capacitance (C_{dl}), CV was collected in pre-OER potential region at various scan rates from 40 to 120 mV s^{-1}.

Characterization. Scanning electron microscopy (SEM) was characterized with an accelerating voltage of 10 kV by Hitachi S-8200. The transmission electron microscopy (TEM) and high-resolution transmission electron microscopy (HRTEM) images were performed on JEM-F200 microscope. The X-ray diffraction (XRD) patterns were operated on X'Pert PRO MPD with a 2θ range from 5 to 90°at a scan rate of 1 °per min. The valence states of the prepared samples was analyzed by X-ray photoelectron spectroscopy (XPS) using AXIS SUPRA with an Al *K*α source. The XPS spectra were calibrated by C 1s spectra, and its main line was set to 284.6 eV. The existence of P defects were confirmed with electron paramagnetic resonance

(EPR) by Bruker A300 Germany. Raman spectroscopy was recorded on the mentioned Renishaw Raman microscope under controlled potentials by an electrochemical workstation with an excitation of 532 nm laser.

Determination of the PZC. The point of zero charge (PZC) for Cr_2O_3-NiFeOOH and NiFeOOH electrodes was evaluated using differential capacitance minima in a three-electrode configuration, employing a Pt plate counter electrode and Hg/HgO (1.0 M KOH) reference electrode. Impedance-potential profiles were acquired at 1 Hz frequency with 5 mV AC amplitude and 5 mV potential steps. To investigate anion influences, PZC measurements were conducted in KOH and KCl solutions across a concentration range (10-100 mM). The PZC values were identified from the specific capacitance (C_d) minima, derived from the imaginary impedance component (Z") at 1 Hz angular frequency (ω) using the relation Cd = |1/(ωZ")|.

Colorimetric detection experiment. Quantification of free chlorine species (Cl_2, HClO, and ClO^-) was performed using DPD colorimetric analysis. For each measurement, 20 mL of electrolyte was first acidified to pH 6-7 using dilute HCl, followed by addition of 5 mL phosphate buffer (pH 6.5) and 5 mL DPD reagent solution (1.1 g/L N,N-diethyl-1,4-phenylenediamine sulfate). The DPD reagent undergoes immediate chromogenic reaction with free chlorine, producing the characteristic Worcester dye. UV-Vis spectroscopy at 550 nm was employed to measure the absorbance of the resulting solution. This analytical procedure was applied to both alkaline seawater electrolytes containing varying ClO^- concentrations and post-electrolysis samples obtained after 500 h operation at 100 mA cm^{-2}.

Calculations of AEM electrolyzer efficiency and H_2 cost. These calculations only considered the electricity costs, based on the method proposed by literature:

H_2 production rate@0.1 A cm^{-2}

$= (j\ A\ cm^{-2})(1\ e^-/1.602 \times 10^{-19}\ C)(1\ H_2/2\ e^-)$

$= 0.1\ A\ cm^{-2} / (1.602 \times 10^{-19}\ C \times 2) = 5.18 \times 10^{-7}\ mol\ H_2\ cm^{-2}\ s^{-1}$

LHV of H_2

$= 120\ kJ\ g^{-1}\ H_2 = 2.42 \times 10^5\ J\ mol^{-1}\ H_2$

H_2 power out

$= (H_2\ production\ rate) \times (LHV\ of\ H_2)$

$= (5.1828 \times 10^{-7}\ mol\ cm^{-2}\ s^{-1}) \times (2.42 \times 10^5\ J\ mol^{-1}) = 0.1254\ W\ cm^{-2}$

Electrolyzer Power @ 0.1 A cm^{-2}

= (j A cm^{-2}) × (Potential V)
= (0.1 A cm^{-2}) × (1.511 V) = 0.12536 W cm^{-2}

Efficiency of AEM
= (H_2 Power Out) / (Electrolyzer Power)
= 0.1254 W cm^{-2}/0.12536 W cm^{-2} = 83.0%

Price per gasoline-gallon equivalent (GGE) H_2
= 1 GGE H_2/H_2 production rate × Electrolyzer power × Electricity bill
= 0.997 kg / (5.1828 × 10^{-7} mol H_2.cm^{-2} s^{-1} × 2 kg/mol) × 0.12536 W cm^{-2} × \$ 0.02 / kW h = \$ 0.81 /GGE H_2

XPS and XAFS measurements with different applied electrode potentials. Electrochemical measurements at different potentials were carried out using a standard three-electrode configuration, with the as-prepared pre-catalyst, a Hg/HgO electrode, and a graphite rod serving as the working, reference, and counter electrodes, respectively. A sample with dimensions of 1 cm × 1 cm was subjected to a series of anodic potentials ranging from 1.2 V to 1.6 V (vs. RHE), each held for 600 s to ensure steady-state conditions. Then, ultrasonic separation of the sample from the substrate, followed by centrifugation and vacuum drying to obtain powder. Sufficient powder was collected through this procedure, and a small amount of residual NF substrate impurities was removed prior to subsequent characterizations. The valence states of the prepared samples was analyzed by X-ray photoelectron spectroscopy (XPS) using AXIS SUPRA with an Al *K*α source. The XAFS data (Ni K3-edge) was carried out using the RapidXAFS 2M (Anhui Absorption Spectroscopy Analysis Instrument Co., Ltd.) by transmission (or fluorescence) mode at 20 kV and 20 mA. The sample data of Ni_2O_3 and Ni foil were both derived from the standard sample data.

Computational details. All first-principles calculations were conducted using the density functional theory (DFT) technique through the Vienna ab initio simulation package (VASP). Spin-polarized calculations were performed using the generalized gradient approximation (GGA) combined with the Perdew-Burke-Ernzerhof (PBE) method to determine the exchange and correlation energies. The PAW pseudopotentials treated core electrons while expanding valence states in a plane-wave basis with a 400 eV cutoff energy. The DFT+U method was applied to correct the transition metals, with U values of 3.0 eV for Cr, 5.0 eV for Fe, and 5.5 eV

for Ni [1-3]. Electronic states were smeared using Gaussian distributions ($\sigma = 0.05$ eV) until achieving self-consistency ($\Delta E < 10^{-5}$ eV). Structural relaxations proceeded until atomic forces fell below 0.03 eV/Å, with van der Waals corrections incorporated via DFT-D3. The Brillouin zone integration employed 2 × 2 × 1 Monkhorst-Pack grids.

The model establishment of NiFeOOH first integrated XRD and HRTEM, and determined that the crystal plane of NiFeOOH was the (001) crystal plane of NiOOH. And a vacuum of 15 Å was used to simulate the surface under periodic boundary conditions. According to the article, we have determined that the model of γ-NiOOH is the NiOOH structure of COD IPD-9012318 in the crystal database. The model of β-NiOOH corresponds to the mp-999337 NiOOH structure in the crystal database. Based on the analysis results of the atomic ratio of Ni to Fe on the surface by XPS, it was determined that the ratio of Ni to Fe was 2:1. Thus, the corresponding amount of Ni in NiOOH was replaced by Fe. The structure of Cr_2O_3 on the surface of Cr_2O_3-NiFeOOH was also referred to the results of XRD and HRTEM. To more intuitively demonstrate the interaction between Cr_2O_3 and NiFeOOH, we established a relatively small Cr_2O_3 crystal with exposed crystal planes of (113) and placed it on the surface of NiFeOOH. Through dynamic calculations, Cr_2O_3 was stably loaded on the surface of the NiFeOOH structure.

The adsorption energy (E_{ads}) the other molecule on the surface was calculated as follows (eq 1):

$$E_{ads}=E_{adsorbate+surface}-E_{surface}-E_{gas} \quad (1)$$

where $E_{surface}$ is the clean surface relaxation energy of the surface slab, E_{gas} is the energy of a free gas molecule under conditions of vacuum, and $E_{adsorbate+surface}$ is the energy of the composite system.

The barrier energy, ΔE, for a difference between transition state energy difference and standard product formation enthalpy was defined as follows. The energy barrier calculation of H_2O to H-OH on NiFeOOH as an example (eq 2):

$$\Delta E=(E_{H-NiFeOOH}-E_{stab-NiFeOOH})+(E_{OH-NiFeOOH}-E_{stab-NiFeOOH})-(E_{H2O-NiFeOOH}-E_{stab-NiFeOOH}) \quad (2)$$

where $E_{H\text{-}NiFeOOH}$, $E_{OH\text{-}NiFeOOH}$ and $E_{H2O\text{-}NiFeOOH}$ are the energies of the H, OH and H_2O adsorbed on NiFeOOH, $E_{stab\text{-}NiFeOOH}$ is the energy of the NiFeOOH structure surface slab.

As the calculations were performed a t =0 K at a fixed cell volume, the differences in the Gibbs free energy should equal the differences in the total energy. By this definition, the lower the ΔE is, the easier it is to react.

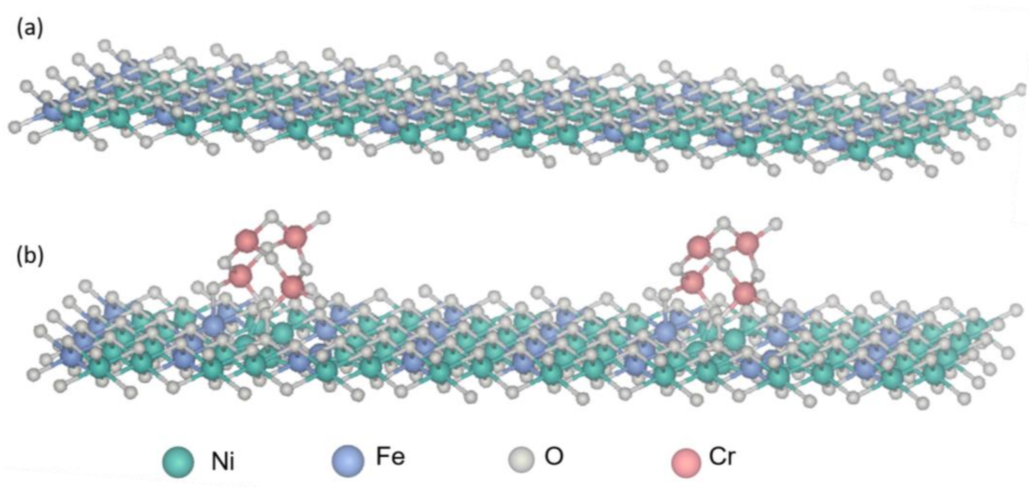

Fig. S1 Strueture models of (a) NiFeOOH and (b) Cr_2O_3-NiFeOOH.

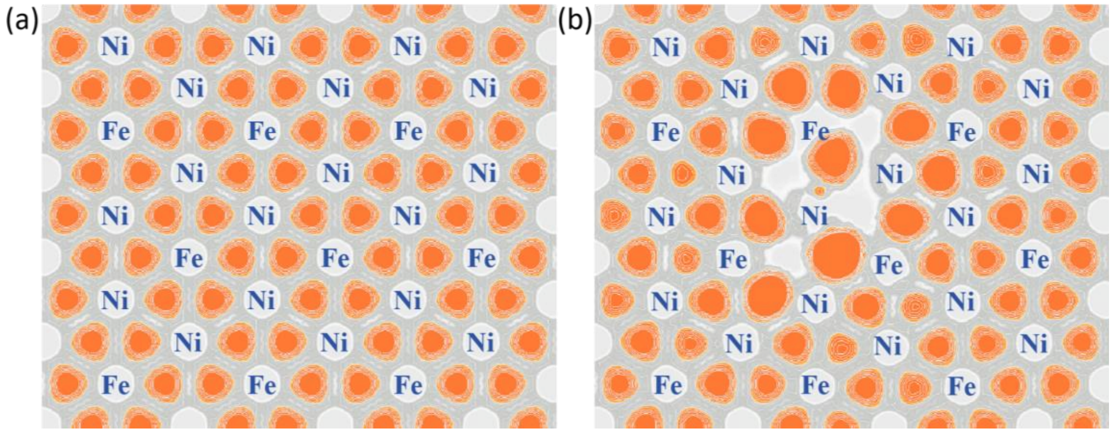

Fig. S2 Electron Locational Function plot for (a) NiFeOOH and (b) Cr_2O_3-NiFeOOH.

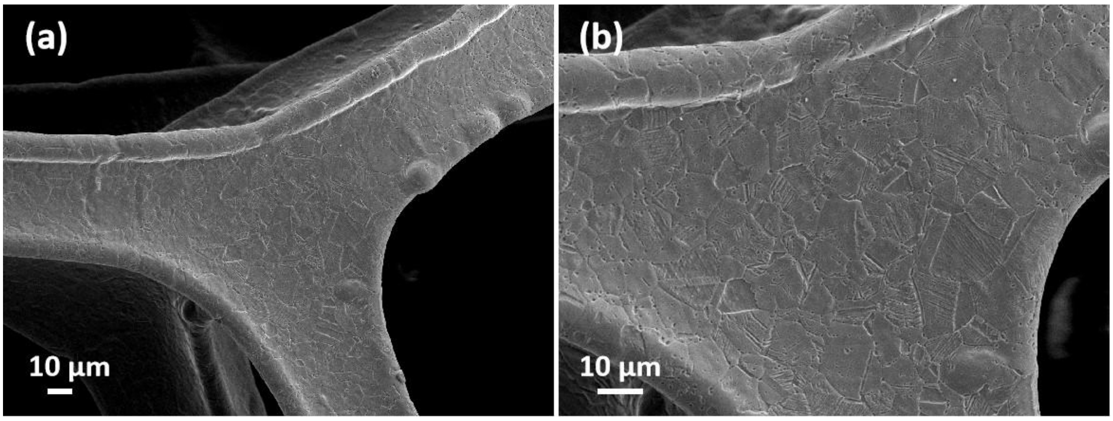

Fig. S3 (a,b) SEM images of NF.

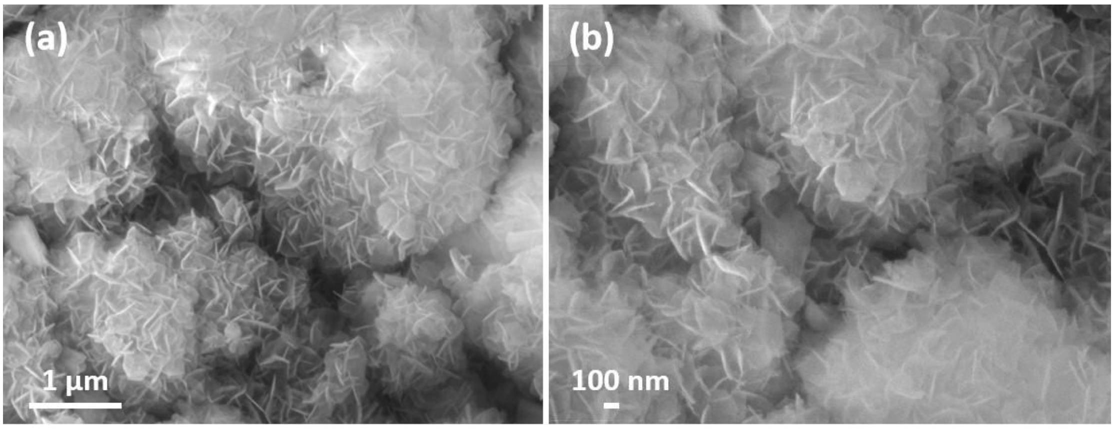

Fig. S4 (a,b) SEM images of Cr_2O_3-NiFe-LDH.

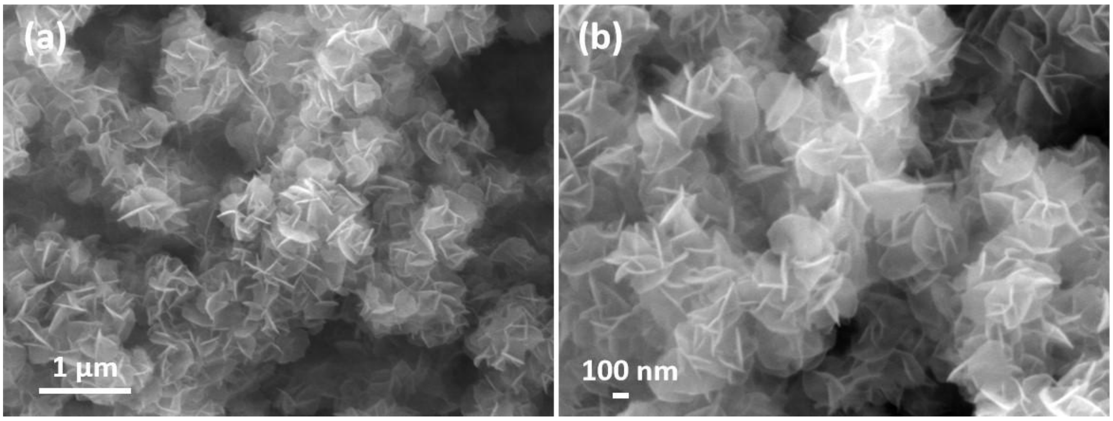

Fig. S5 (a,b) SEM images of NiFe-LDH.

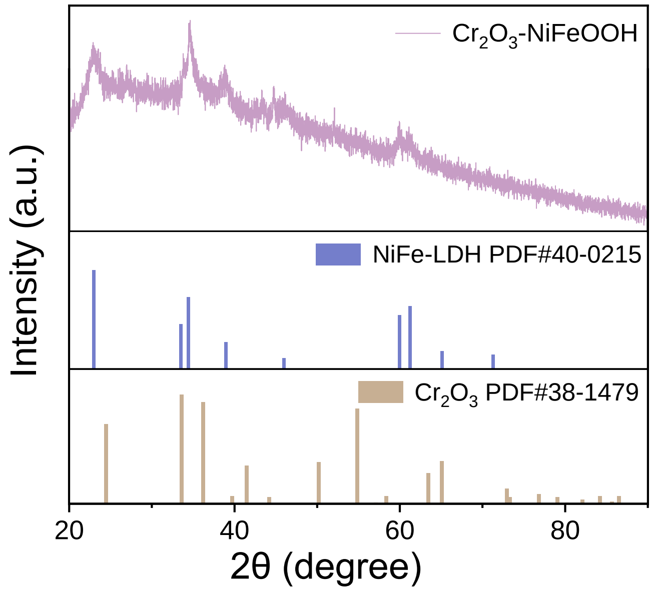

Fig. S6 XRD image of Cr_2O_3-NiFeOOH.

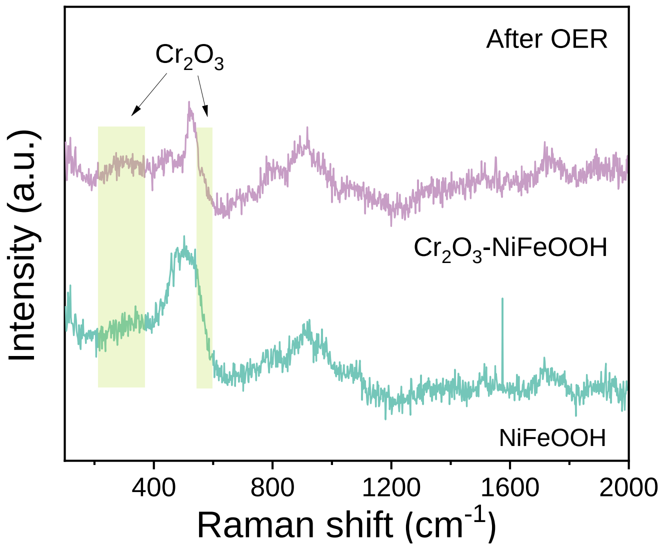

Fig. S7 Raman spectra of Cr_2O_3-NiFeOOH and NiFeOOH.

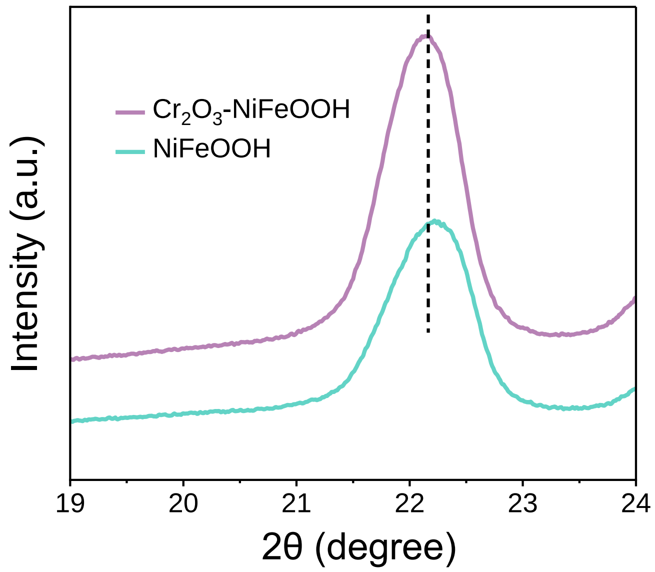

Fig. S8 XRD image of Cr_2O_3-NiFeOOH and NiFeOOH after activation in electrolytes.

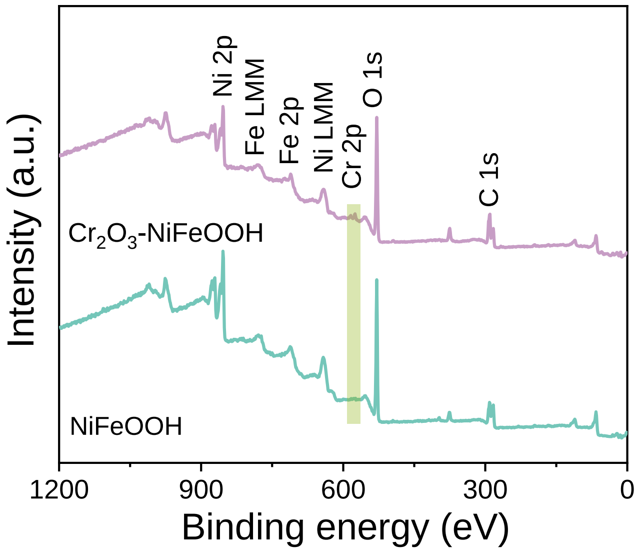

Fig. S9 XPS survey spectra of Cr_2O_3-NiFeOOH and NiFeOOH.

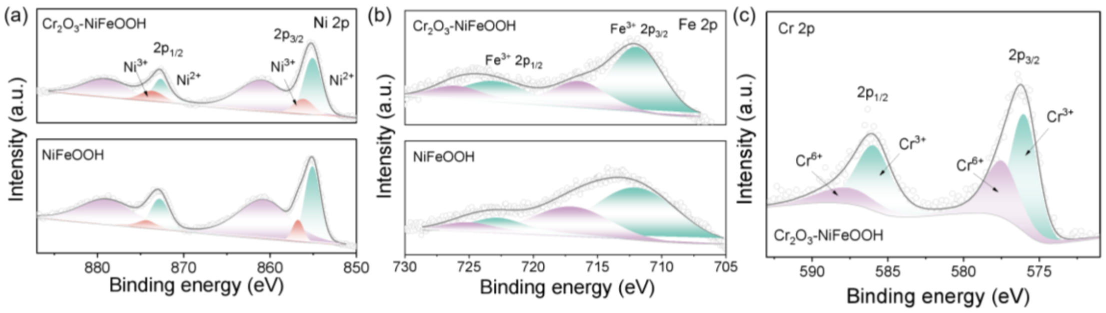

Fig. S10 High-resolution of (a) Ni 2p XPS spectra, (b) Fe 2p XPS spectra, and (c) Cr 2p XPS spectra of Cr_2O_3-NiFeOOH and NiFeOOH after OER.

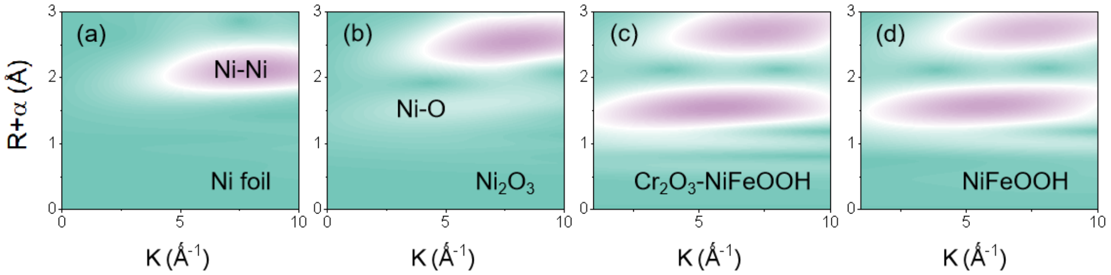

Fig. S11 The wavelent transform plots of (a) Ni foil, (b) Ni_2O_3, (c) Cr_2O_3-NiFeOOH and (d) NiFeOOH.

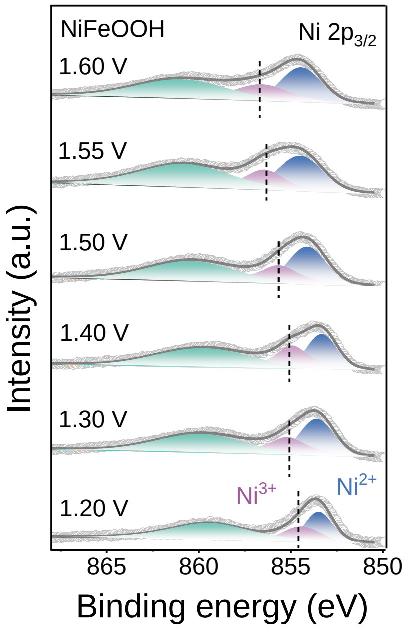

Fig. S12 The quasi in-situ XPS spectra of Ni 2p for NiFeOOH under operando potentials.

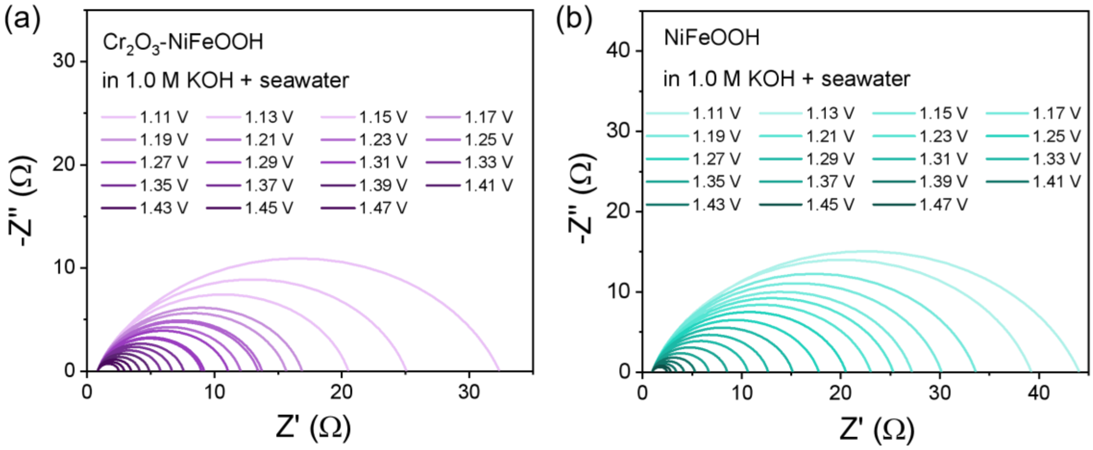

Fig. S13 Nyquist plots for (a) Cr_2O_3-NiFeOOH and (b) NiFeOOH at different applied potentials for OER.

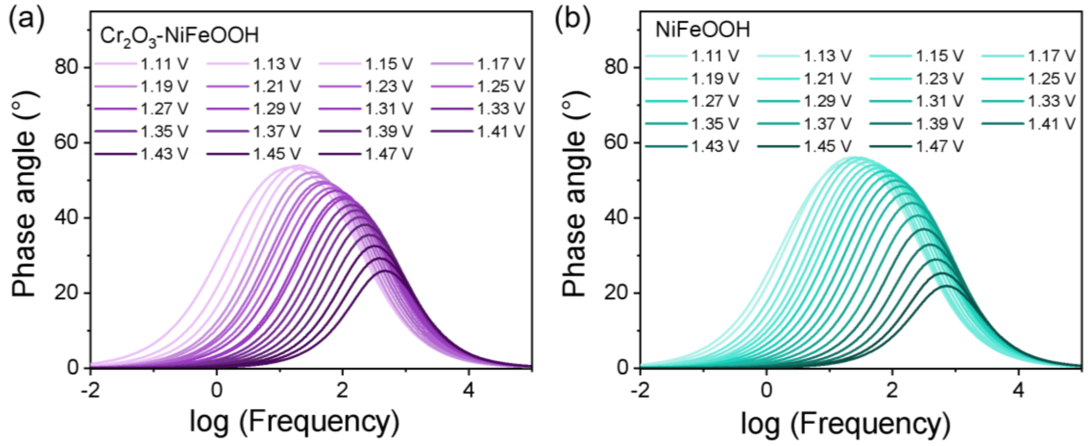

Fig. S14 Bode-phase plots of (a) Cr_2O_3-NiFeOOH and (b) NiFeOOH under different operated potentials (vs. RHE).

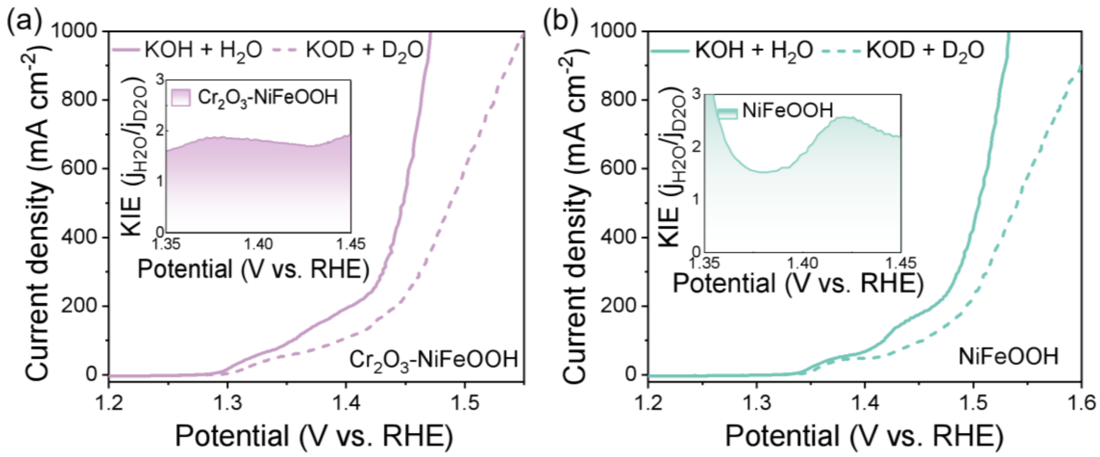

Fig. S15 LSV curves of (a) Cr_2O_3-NiFeOOH and (b) NiFeOOH in 1.0 M KOH/H_2O solution and 1.0 M KOD/D_2O solution, the inset represents the kinetic isotope effect values (KIEs =$j_{H2O/D2O}$) vs. Potential.

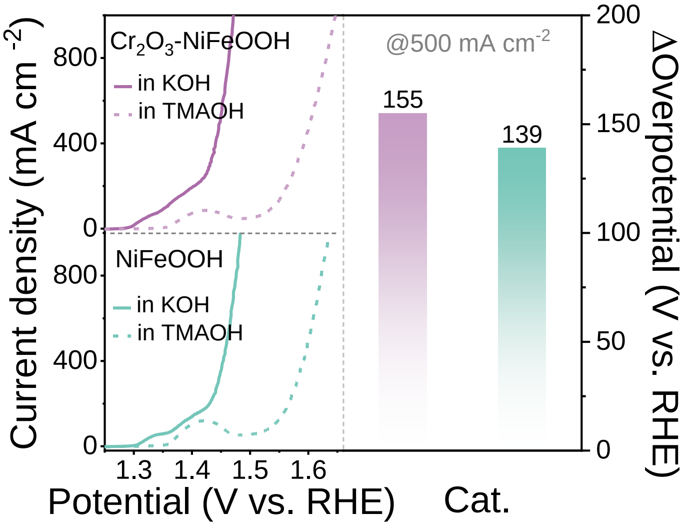

Fig. S16 LSV curves in 1.0 M KOH and TMAOH.

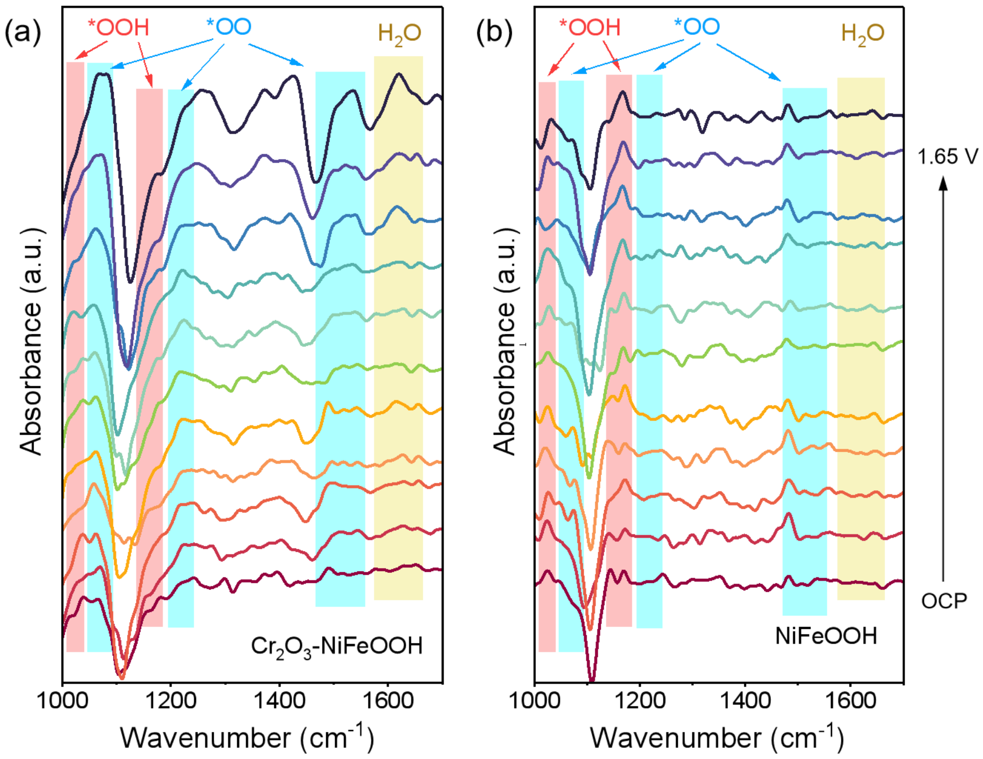

Fig. S17 In situ ATR-SEIRAS collected from OCP to 1.65 V vs. RHE for Cr_2O_3-NiFeOOH and NiFeOOH.

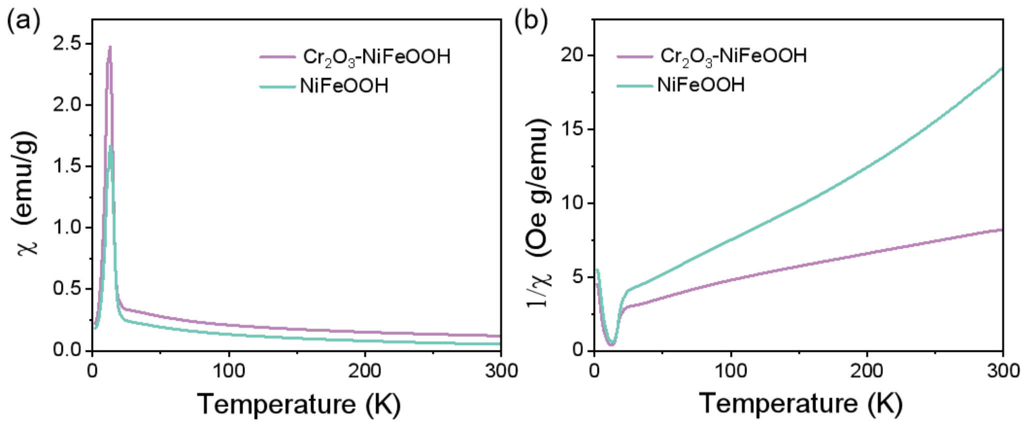

Fig. S18 (a) Temperature-dependent susceptibility χ and (b) temperature-dependent inverse susceptibility 1/χ of Cr_2O_3-NiFeOOH and NiFeOOH.

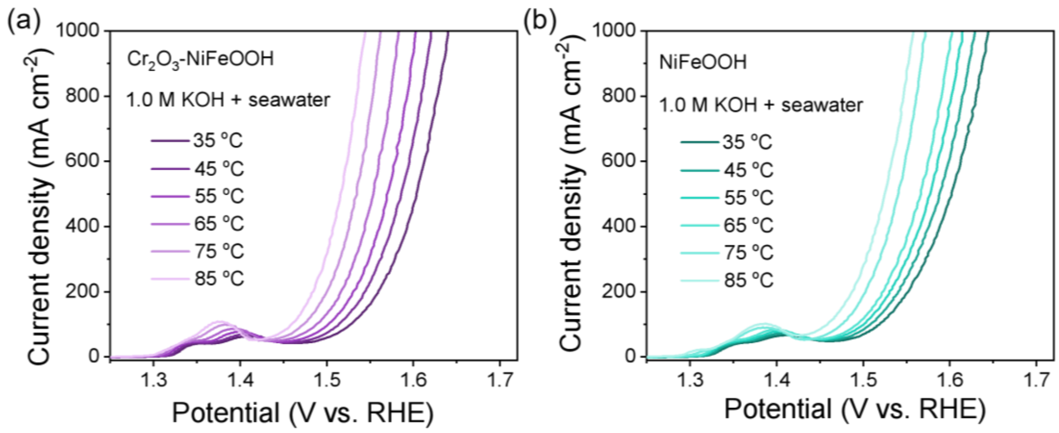

Fig. S19 LSV curves of (a) Cr_2O_3-NiFeOOH and (b) NiFeOOH in 1.0 M KOH+seawater solution at different temperatures.

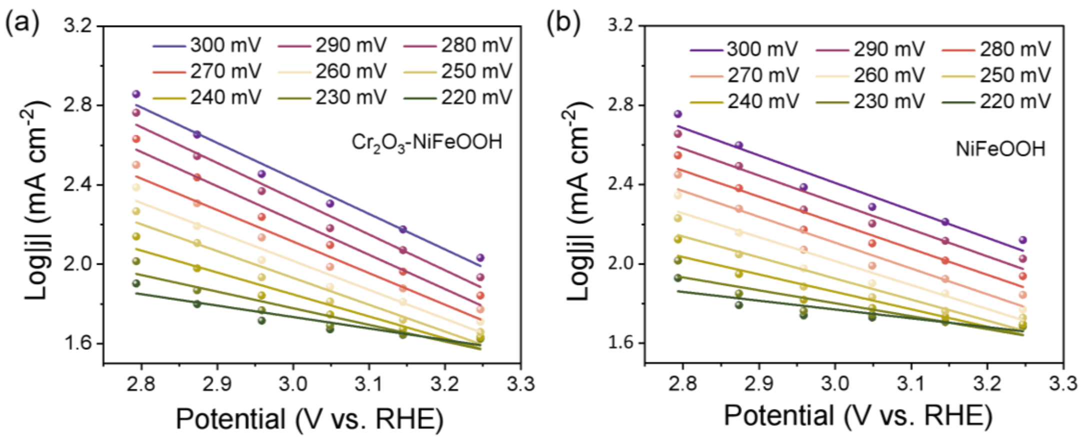

Fig. S20 The logarithm of the catalytic current density plotted against 1000 times the reciprocal of the temperature (in Kelvin) to extract the pre-exponential factor (A_{app}) of the alkaline OER on (a) Cr_2O_3-NiFeOOH and (b) NiFeOOH catalysts at fixed overpotentials using the Arrhenius plots.

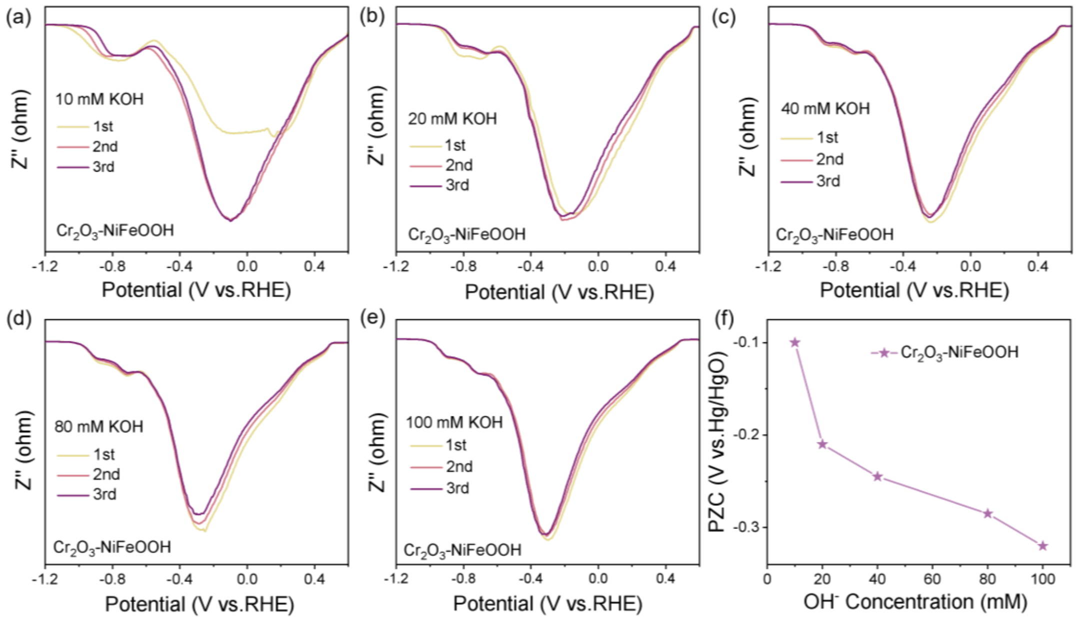

Fig. S21 The electrochemical impedance-potential spectra of Cr_2O_3-NiFeOOH electrodes in 10, 20, 40, 80 and 100 mM KOH solutions, respectively.

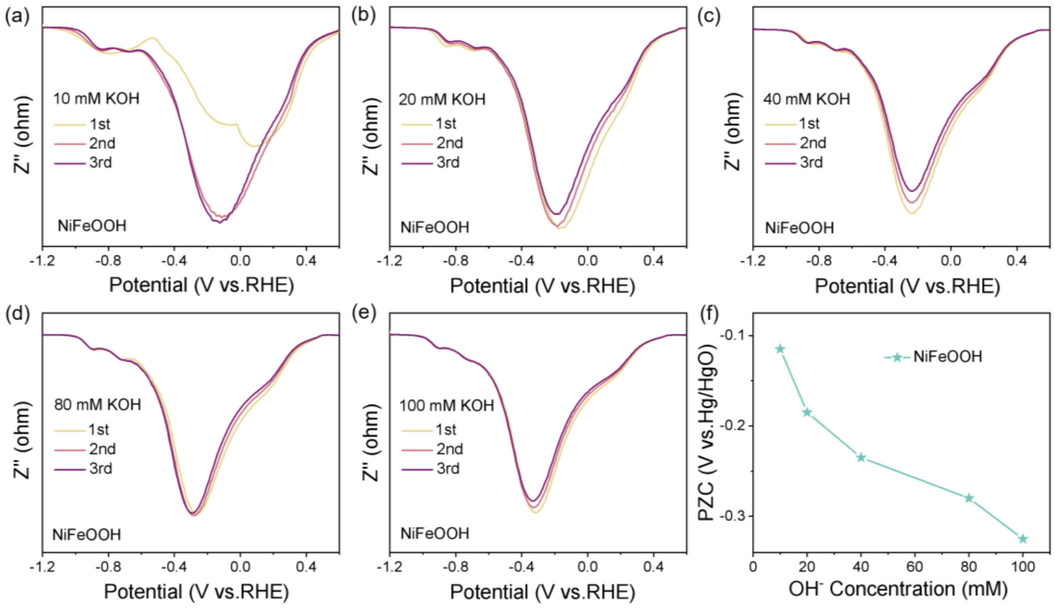

Fig. S22 The electrochemical impedance-potential spectra of NiFeOOH electrodes in 10, 20, 40, 80 and 100 mM KOH solutions, respectively.

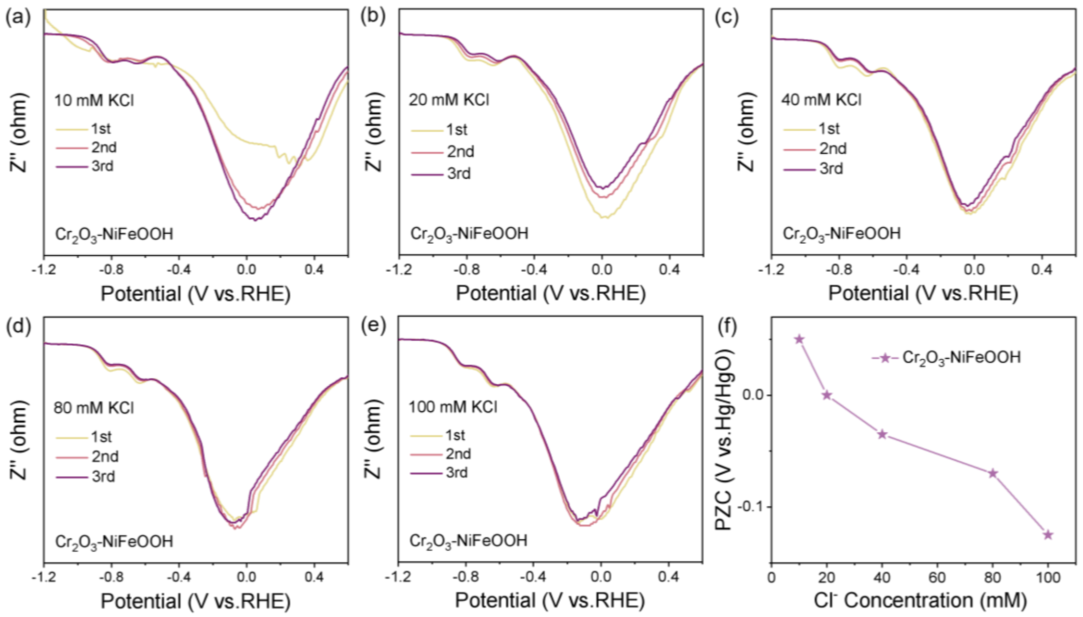

Fig. S23 The electrochemical impedance-potential spectra of Cr_2O_3-NiFeOOH electrodes in 10, 20, 40, 80 and 100 mM KCl solutions, respectively.

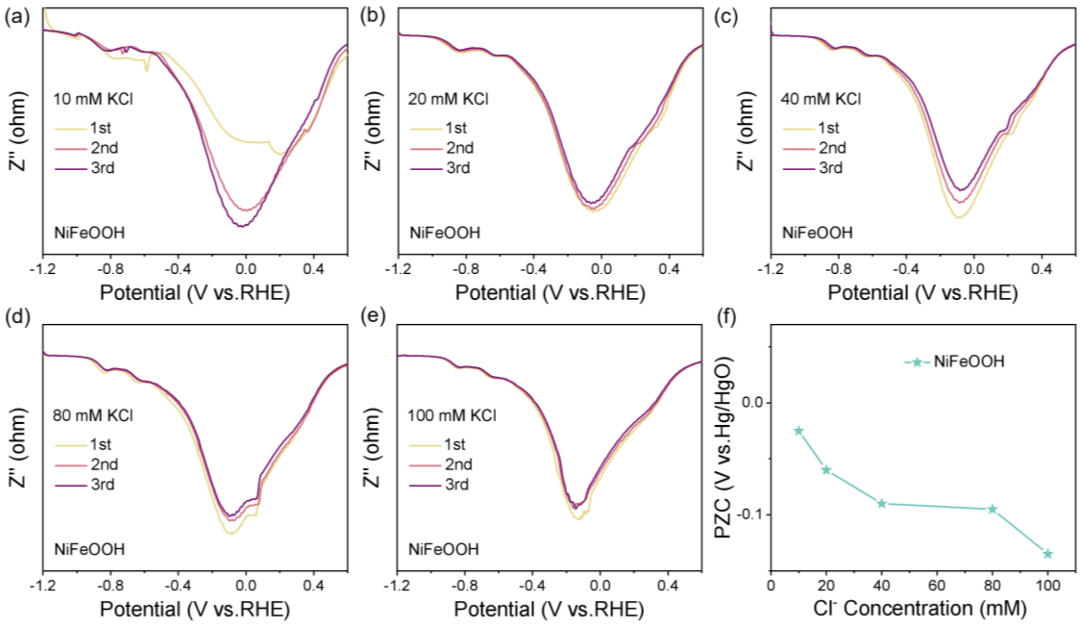

Fig. S24 The electrochemical impedance-potential spectra of NiFeOOH electrodes in 10, 20, 40, 80 and 100 mM KCl solutions, respectively.

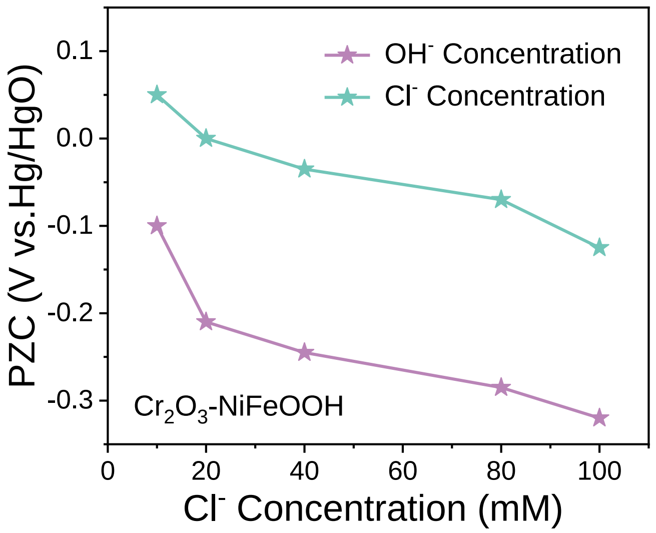

Fig. S25 PZCs of Cr_2O_3-NiFeOOH electrodes in 0, 10, 20, 40, 80, 100 mM KOH and KCl solutions.

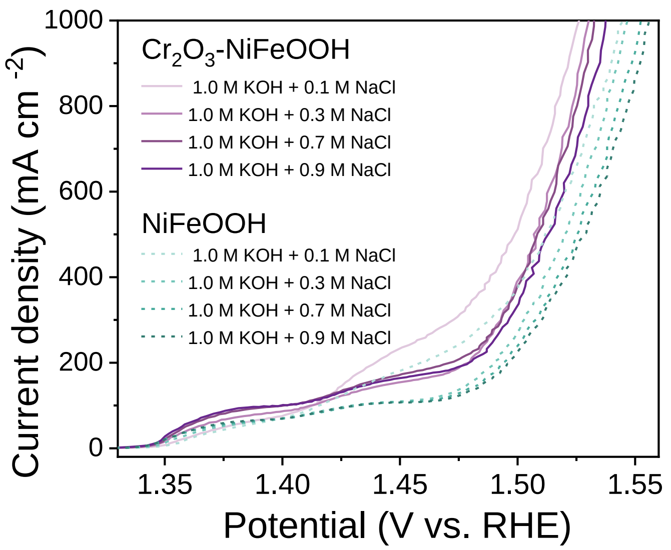

Fig. S26 OER polarization curves of Cr_2O_3-NiFeOOH and NiFeOOH in different Cl^- concentrations.

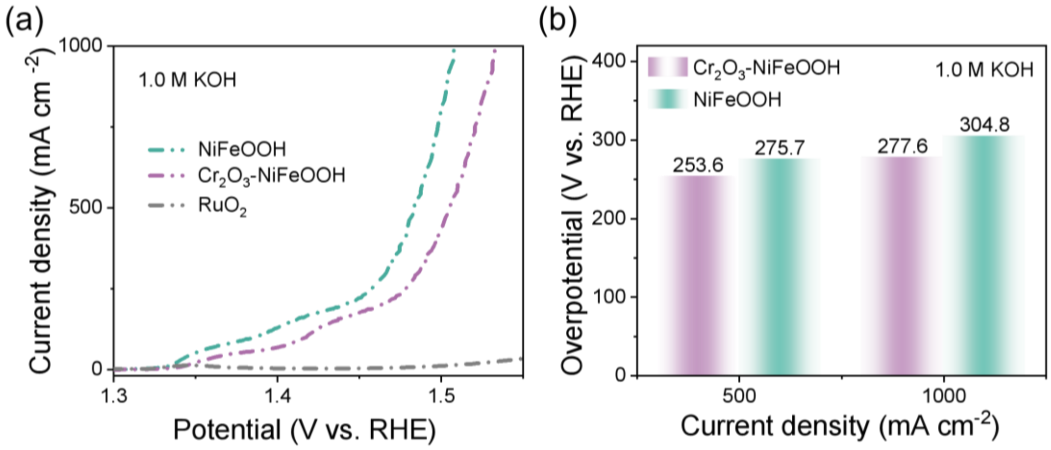

Fig. S27 (a) LSV curves and (b) overpotential of Cr_2O_3-NiFeOOH and NiFeOOH for OER performance in 1.0 M KOH.

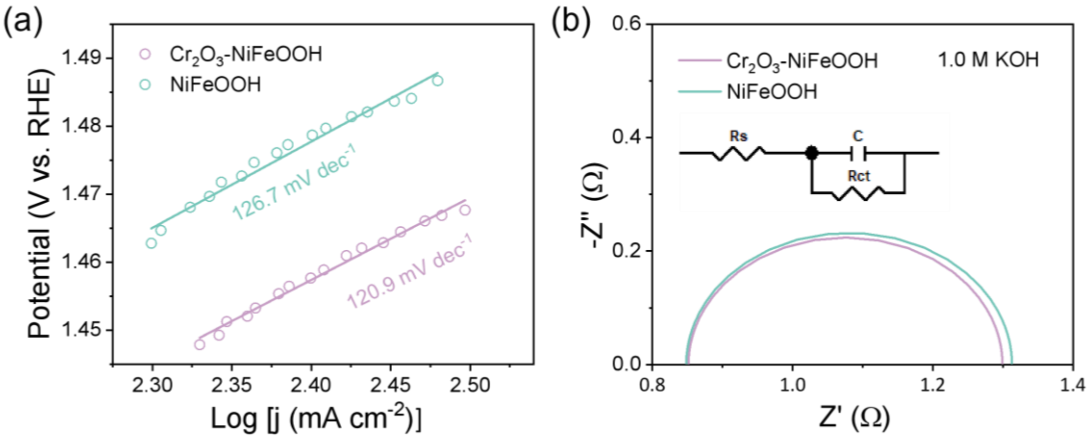

Fig. S28 (a) The Tafel plots and (b) Nyquist plots of Cr_2O_3-NiFeOOH and NiFeOOH for OER performance in 1.0 M KOH.

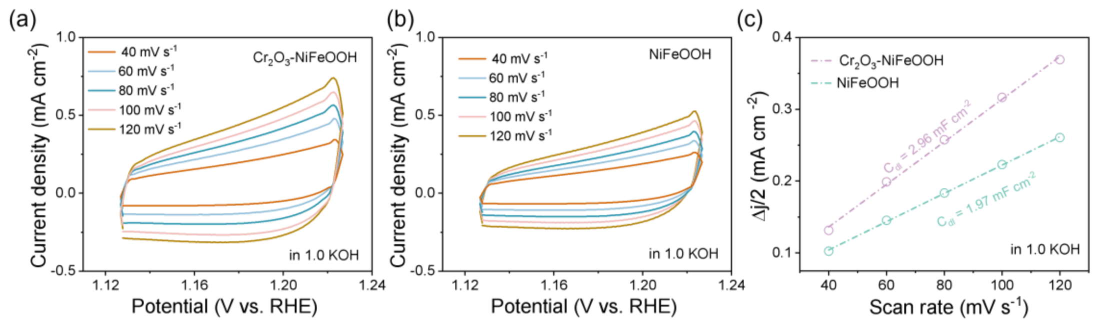

Fig. S29 CV for measuring ECSAs of as-prepared samples: (a) Cr_2O_3-NiFeOOH and (b) NiFeOOH. (c) C_{dl} values of as-prepared samples for OER in 1.0 M KOH.

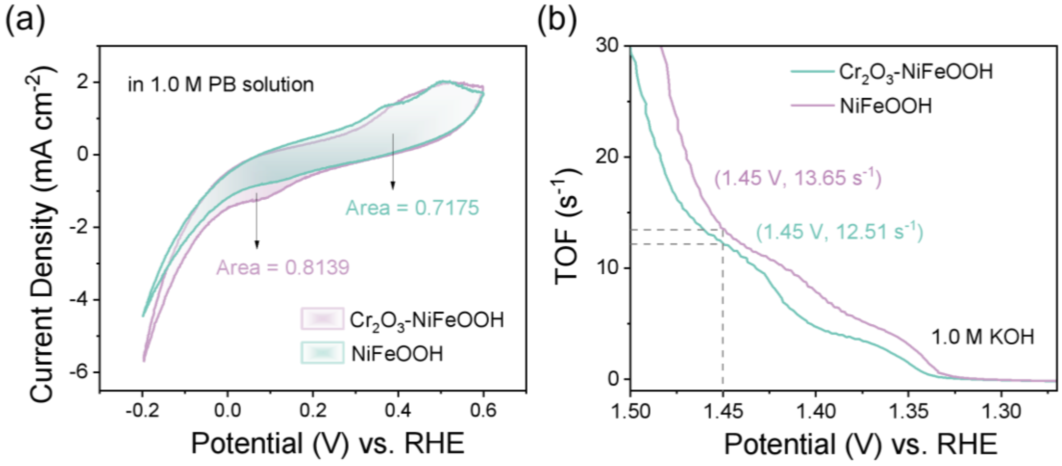

Fig. S30 (a) CVs for Cr_2O_3-NiFeOOH and NiFeOOH in phosphate buffer (pH=7) at a scan rate of 50 mV s^{-1}. (b) Polarization curves of OER normalized by active sites and expressed in terms of TOF in 1.0 M KOH.

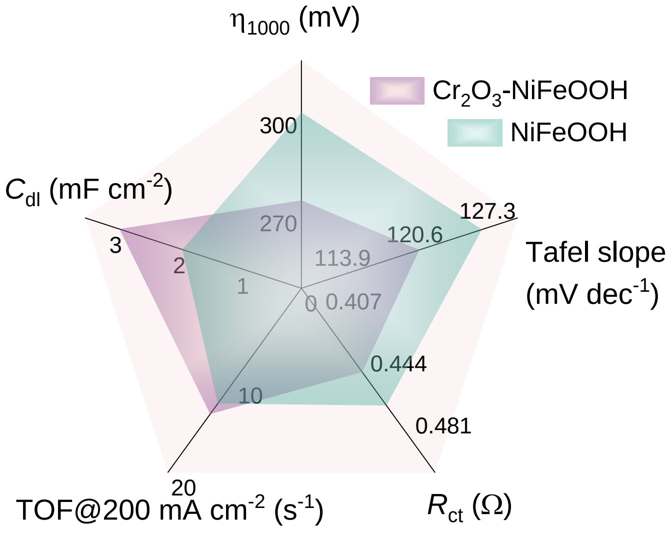

Fig. S31 Some major OER performance metrics of Cr_2O_3-NiFeOOH and NiFeOOH in 1.0 M KOH.

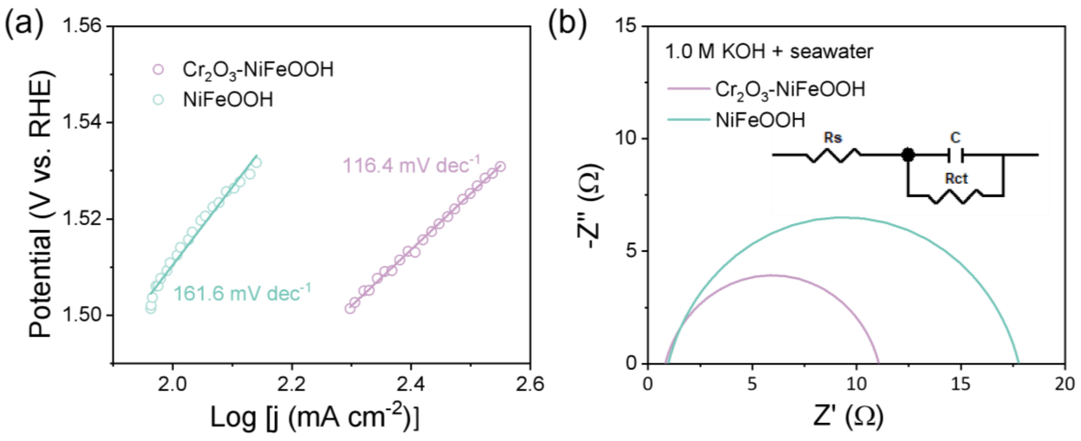

Fig. S32 (a) The Tafel plots and (b) Nyquist plots of Cr_2O_3-NiFeOOH and NiFeOOH for OER performance in 1.0 M KOH + seawater.

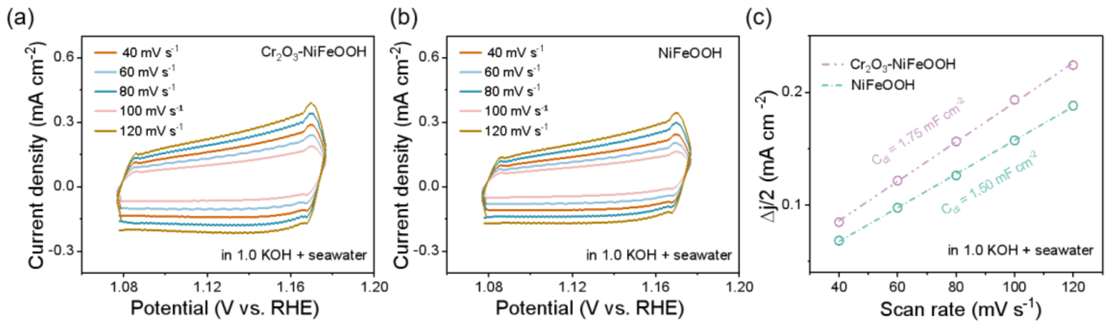

Fig. S33 CV for measuring ECSAs of as-prepared samples: (a) Cr_2O_3-NiFeOOH and (b) NiFeOOH. (c) C_{dl} values of as-prepared samples for OER in 1.0 M KOH + seawater.

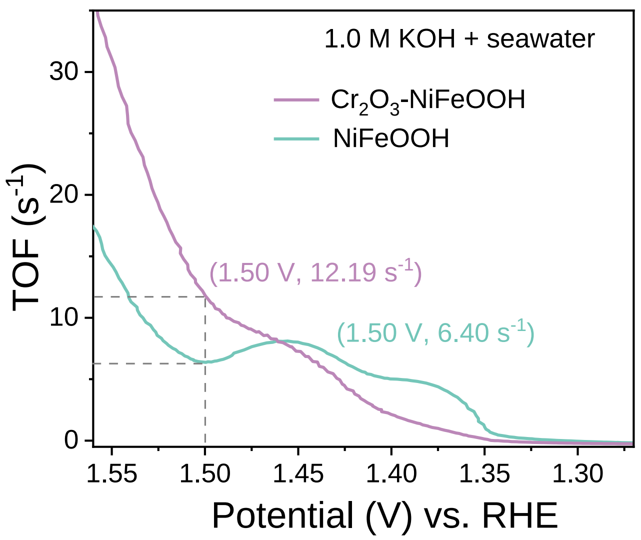

Fig. S34 Polarization curves of OER normalized by active sites and expressed in terms of TOF in 1.0 M KOH + seawater.

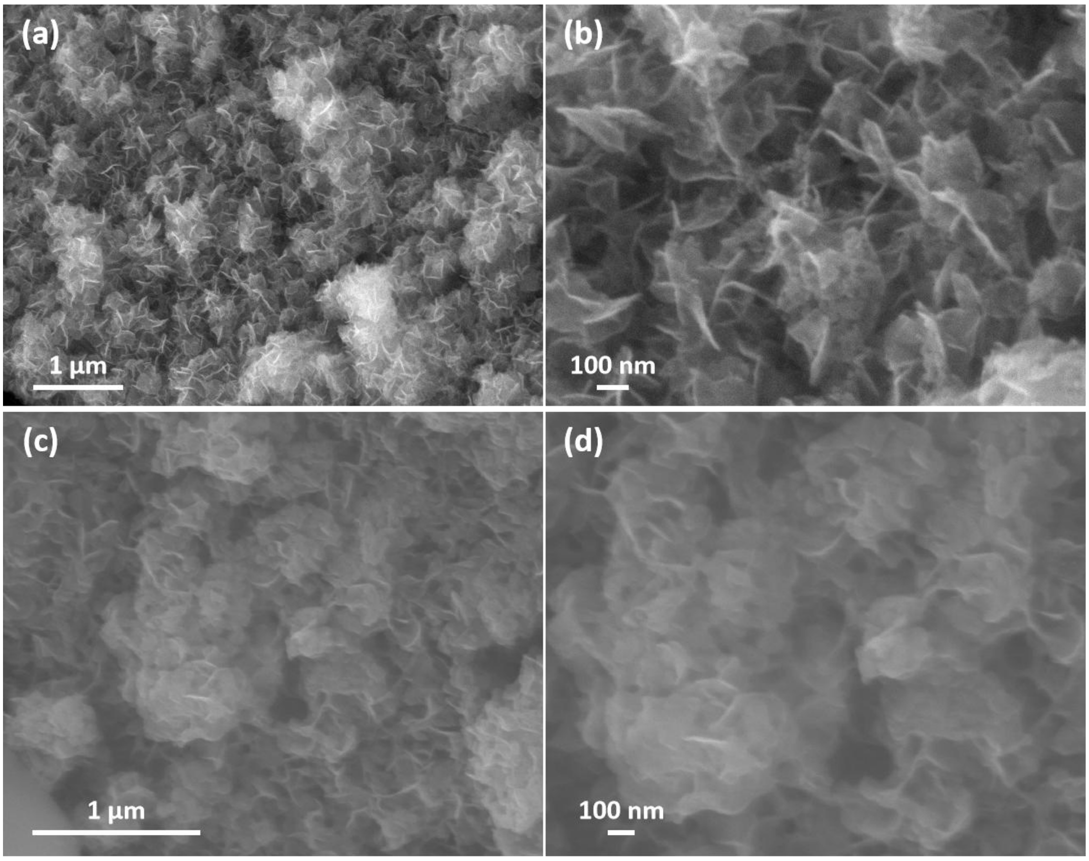

Fig. S35 SEM images of (a-b) Cr_2O_3-NiFeOOH and (c-d) NiFeOOH after durability test for OER.

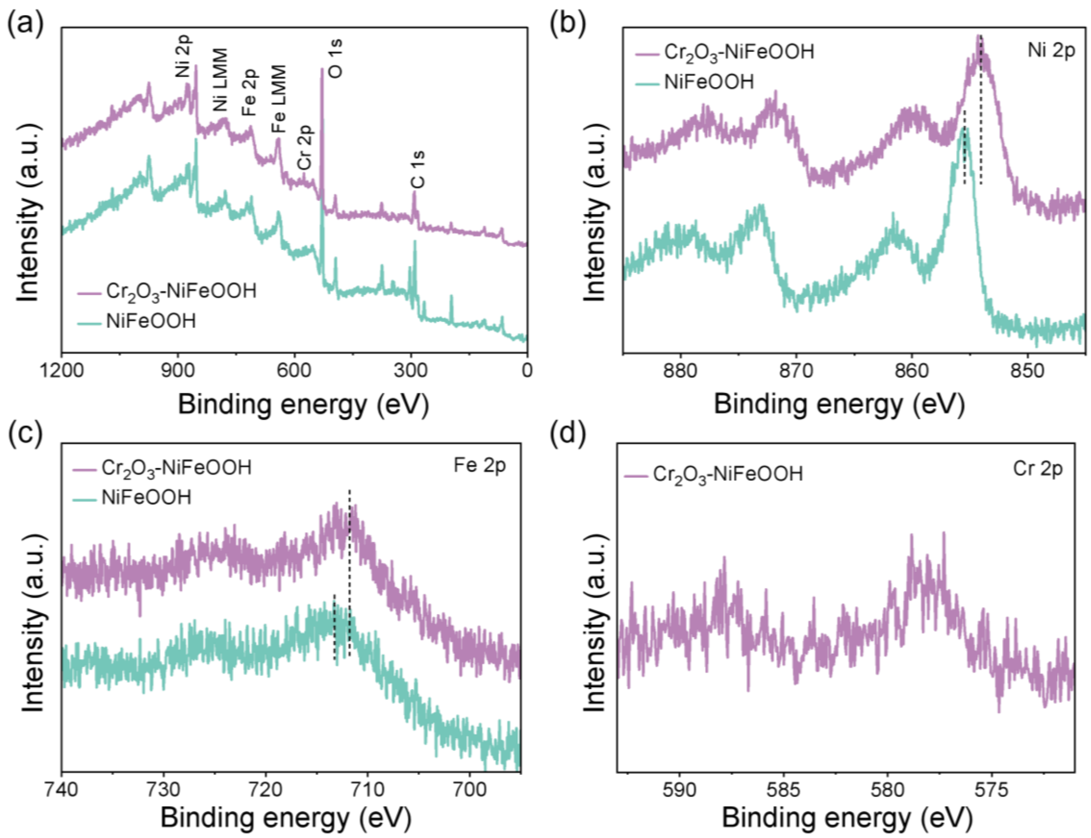

Fig. S36 (a) XPS survey spectra of Cr_2O_3-NiFeOOH and NiFeOOH after durability test for OER. High-resolution of (b) Ni 2p and (c) Fe 2p XPS spectra of Cr_2O_3-NiFeOOH and NiFeOOH after durability test for OER. (d) Cr 2p XPS spectra of Cr_2O_3-NiFeOOH after durability test for OER.

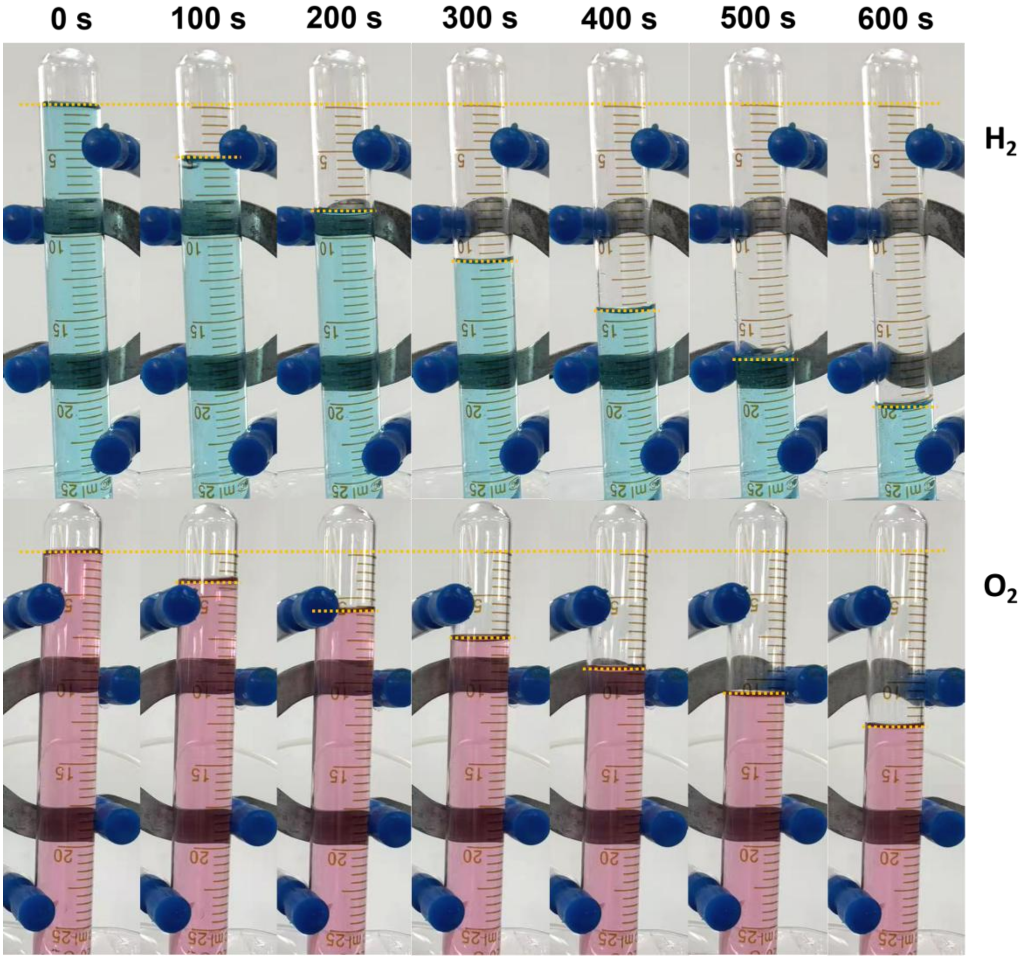

Fig. S37 The corresponding photographs of the gas of H_2 and O_2 varying with time of Cr_2O_3-NiFeOOH at 250 mA cm^{-2} in 1.0 M KOH+seawater.

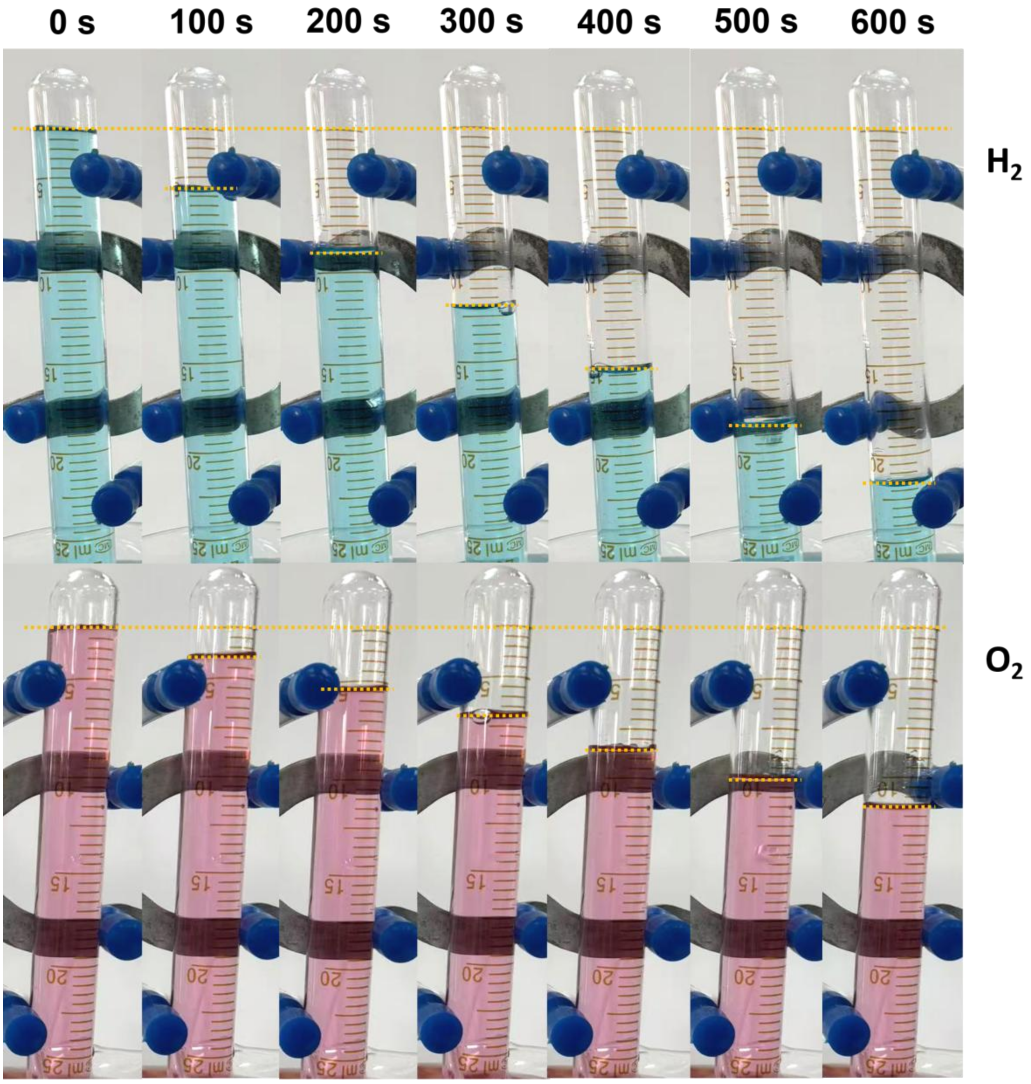

Fig. S38 The corresponding photographs of the gas of H_2 and O_2 varying with time of NiFeOOH at 250 mA cm^{-2} in 1.0 M KOH+seawater.

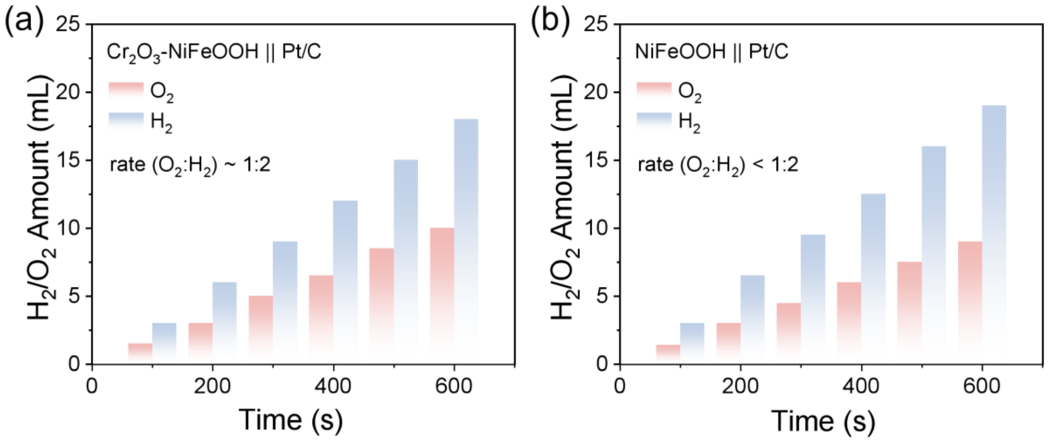

Fig. S39 Comparison between the amount of collected gaseous products of (a) Cr_2O_3-NiFeOOH and (b) NiFeOOH for overall seawater splitting.

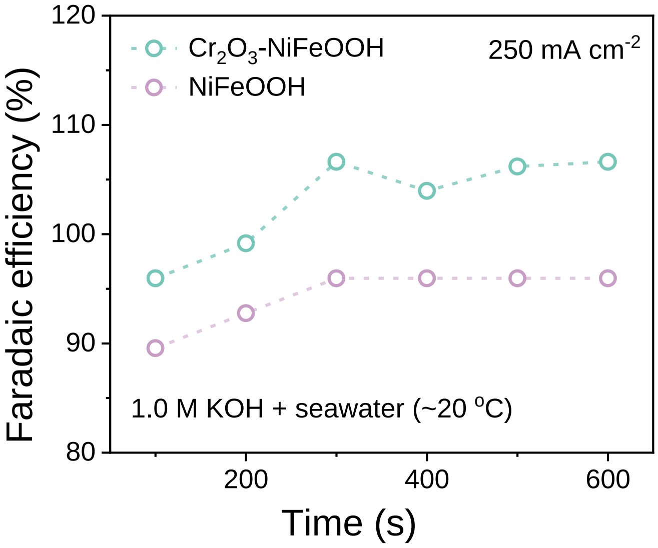

Fig. S40 Comparison of Faraday efficiencies of Cr_2O_3-NiFeOOH and NiFeOOH.

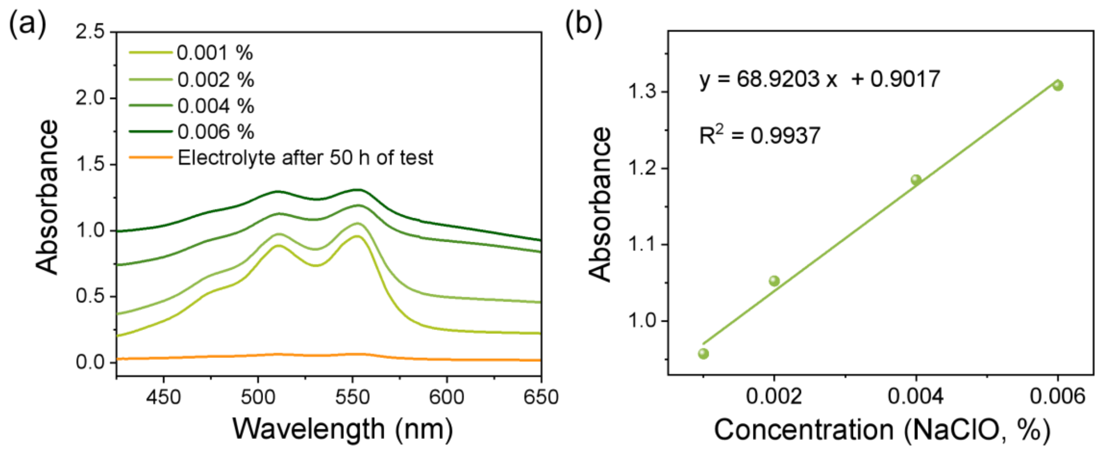

Fig. S41 (a) UV-Vis absorption spectra of ClO^- with different concentrations. (b) Calibration curve used for calculating ClO^- concentrations of the electrolyte.

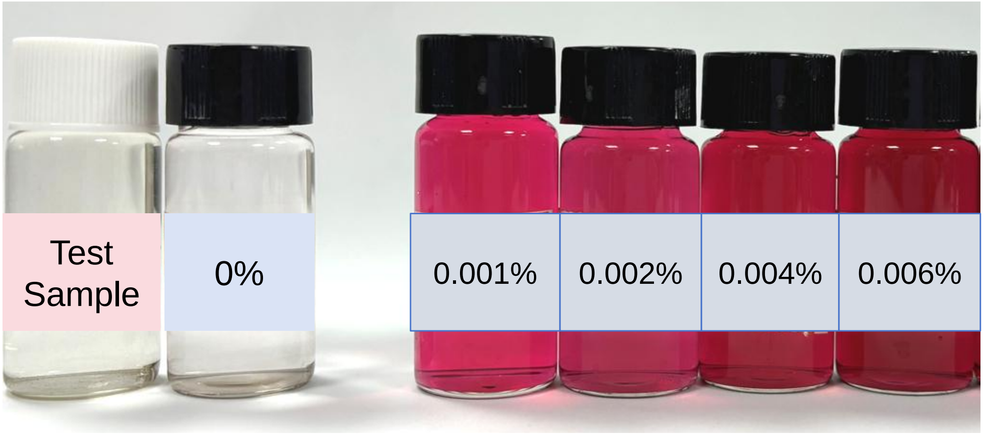

Fig. S42 Hypochlorite detection result for 1.0 M KOH+seawater with different ClO^- contents and the electrolyte after 50 h electrolysis for Cr_2O_3-NiFeOOH||Pt/C at a constant current density of 100 mA cm^{-2} in 1.0 M KOH+seawater.

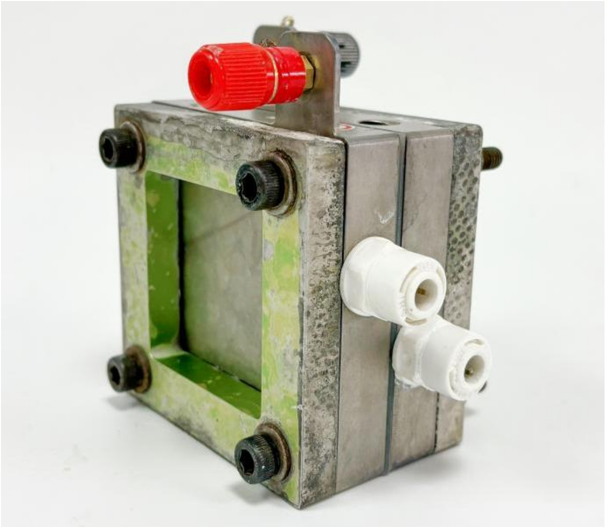

Fig. S43 Optical photo of AEM electrolyzer.

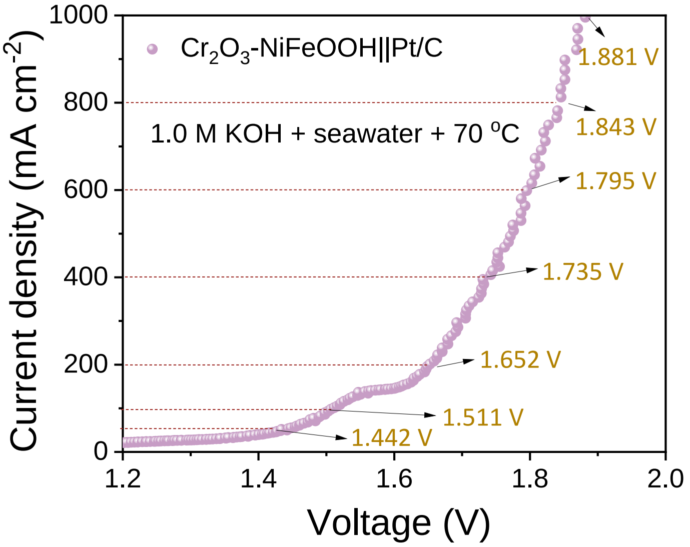

Fig. S44 Polarization curves of the Cr_2O_3-NiFeOOH||Pt/C.

Table S1 Comprehensive comparisons of the OER performance of Cr_2O_3-NiFeOOH with NiFeOOH in 1.0 M KOH.

Catalysts	Potential @1000 (V)	Tafel slope (mV dec^{-1})	R_{ct} (Ω)	TOF (s^{-1})	C_{dl} (mF cm^{-2})
**Cr_2O_3-NiFeOOH**	277	120.9	0.44	13.65	2.96
**NiFeOOH**	304	126.7	0.46	12.51	1.97

TOF (s^{-1}) at overpotential η=1.45 V

Table S2 Comprehensive comparisons of the OER performance of Cr_2O_3-NiFeOOH with NiFeOOH in 1.0 M KOH + seawater.

Catalysts	Potential @500 (V)	Tafel slope (mV dec^{-1})	R_{ct} (Ω)	TOF (s^{-1})	C_{dl} (mF cm^{-2})
**Cr_2O_3-NiFeOOH**	320	157.8	7.56	12.19	1.75
**NiFeOOH**	368	235.7	8.15	6.40	1.50

TOF (s^{-1}) at overpotential η=1.5 V

Table S3 Comparison of potential (η_{500}) of Cr_2O_3-NiFeOOH with other reported materials in AEM electrolyzer at alkaline seawater.

**Catalyst**	**Electrolyte**	**η_{500} for OER (mV)**	**Reference**
**Cr_2O_3-NiFeOOH**	1 M KOH+seawater	1.77	This work
NFCP	1 M KOH+seawater	2.10	*ACS Cata*. **14**, 18322-18332 (2024)
P-OsNiFe	1 M KOH+seawater	1.86	*Adv. Funct. Mater*. **34**, 2408517 (2024)
(Ni,Fe)O(OH)@NiCoS NAs/NF	1 M KOH+seawater	1.91	*J. Energy Chem*, **91**, 370-382 (2024)
$Zn_{0.5}$Fe/NF	1 M KOH+seawater	1.92	*Chem. Eng. J*. **487**, 150253 (2024)
B,V-Ni_2P	1 M KOH+seawater	1.78	*Small* **19**, 2208076 (2023)
Pt QDs@Ni_3N-MoN/Ti	1 M KOH+seawater	2.15	*Adv. Funct. Mater*. **34**, 2403863 (2024)
Mo_2C/NC@0.5Ni	1 M KOH+seawater	1.92	*J. Alloys Compd*. **968**, 172111 (2023)
Cr_2O_3-CoO_x	1 M KOH+seawater	1.78	*Nat. Energy* **8**, 264-272 (2023)
$Ni_3FeN@PO_4^{3-}$/NF	1 M KOH+seawater	1.91	*Adv. Mater.* **37**, 2475427 (2024)
r-Ru-Ni/NiO	1 M KOH+seawater	1.84	*J. Colloid Interface Sci.* **664**, 704-715 (2024)
NiSe/Fe-$Ni(OH)_2$	1 M KOH+seawater	1.95	*Sci. China Chem*. **67**, 3468-3481 (2024)
$Ni_3S_4@Ni(OH)_2$	1 M KOH+seawater	2.15	*J. Colloid Interface Sci.* **654**, 66-75 (2024)
Co_2P–$Ni_{12}P_5$/NF	1 M KOH+seawater	1.81	ACS Sustain. Chem. Eng. **10**, 9956-9968 (2022)

Table S4 Calculations of AEM electrolyzer efficiency and H_2 cost at different current densities in 1.0 M KOH+seawater.

j (mA cm^{-2})	Voltage (V)	H_2 production rate (mol H_2 cm^{-2} s^{-1})	H_2 power out (W cm^{-2})	Electrolyzer Power (W cm^{-2})	Efficiency of AEM (%)	Price per GGE H_2 ($)
50	1.442	2.59E-7	0.06268	0.0721	86.93204	0.77095
100	1.511	5.18E-7	0.12536	0.1511	82.96228	0.80784
200	1.652	1.036E-6	0.25071	0.3304	75.88136	0.88323
400	1.735	2.072E-6	0.50142	0.694	72.2513	0.9276
600	1.795	3.108E-6	0.75214	1.077	69.83621	0.95968
800	1.843	4.144E-6	1.00285	1.4744	68.01736	0.98534
1000	1.881	5.18E-6	1.25356	1.881	66.64327	1.00566

References

1. Tang M, Ge Q. Mechanistic Understanding on oxygen evolution reaction on γ-FeOOH (010) under alkaline condition based on DFT computational study. *Chin J Catal* 2017; **38**: 1621-1628.

2. Jin J, Sun N, Hu W *et al.* Insight into room-temperature catalytic oxidation of nitric Oxide by Cr_2O_3: a DFT study. *ACS Catal* 2018; **8**: 5415-5424.

3. Fidelsky V, Caspary Toroker M. Engineering band edge positions of nickel oxyhydroxide through facet selection. *J Phys Chem* C 2016; **120**: 8104-8108.
